# Structured multi-view relational learning for drug response prediction

**DOI:** 10.1093/bib/bbag619

**Published:** 2026-11-07

**Authors:** Zengwei Wang, Wanchen Li, Junlin Xu, Yajie Meng, Hao Zhou, Shuting Jin

**Affiliations:** School of Computer Science and Technology, Wuhan University of Science and Technology, 2 Huangjiahuxi Road, Hongshan District, Wuhan 430065, Hubei, China; School of Computer Science and Technology, Wuhan University of Science and Technology, 2 Huangjiahuxi Road, Hongshan District, Wuhan 430065, Hubei, China; School of Computer Science and Technology, Wuhan University of Science and Technology, 2 Huangjiahuxi Road, Hongshan District, Wuhan 430065, Hubei, China; School of Computer Science and Artificial Intelligence, Wuhan Textile University, 1 Yangguang Avenue, Jiangxia District, Wuhan 430200, Hubei, China; School of Computer Science and Technology, Wuhan University of Science and Technology, 2 Huangjiahuxi Road, Hongshan District, Wuhan 430065, Hubei, China; School of Computer Science and Technology, Wuhan University of Science and Technology, 2 Huangjiahuxi Road, Hongshan District, Wuhan 430065, Hubei, China

**Keywords:** drug response prediction, multi-view learning, hypergraph learning, interaction modeling, cell-line relation modeling

## Abstract

Drug response prediction (DRP) requires models that jointly characterize molecular drug structure and heterogeneous cellular states. Although recent deep learning approaches have integrated multi-omics profiles with drug representations, many treat omics views as parallel feature sources or merge them before relational modeling, which can weaken omics-view-specific cell-line relation structures. Here, we propose MulDR, a structured multi-view relational learning framework that preserves omics-view-specific cell-line relation structures before drug-conditioned cell–drug interaction. MulDR constructs separate relation structures for pathway activity, gene expression, mutation, and DNA methylation, learns view-specific relational cell representations, and uses complementary motif-level, geometry-aware molecular graph, and Morgan fingerprint drug representations to condition their fusion. With this design, cell-line similarity remains view-dependent until the interaction stage, rather than being prematurely compressed into a single feature or relation space. Extensive experiments show that MulDR consistently outperforms representative and recent baselines under the same evaluation protocol, reducing root mean square error by 6.07% relative to the strongest baseline. Cold-start evaluations on unseen drugs and cell lines further show that MulDR generalizes to previously unseen entities, while ablation studies show that removing either view-specific relational modeling or drug-conditioned fusion consistently compromises predictive performance. Together, these results suggest that preserving omics-specific cell-line relations before drug-conditioned interaction modeling provides an effective strategy for robust DRP.

## Introduction

Cancer cell lines with similar tissue origins can exhibit markedly different responses to the same anticancer compound because drug sensitivity is shaped by genomic, transcriptomic, epigenomic, and pathway-level cellular states. Drug response prediction (DRP) aims to estimate the pharmacological response of a cell–drug pair from molecular information about both entities [1], and has become a central computational task in cancer pharmacogenomics [2]. The Cancer Cell Line Encyclopedia (CCLE) provides large-scale molecular profiles and pharmacological annotations for cancer cell lines [3], while the Genomics of Drug Sensitivity in Cancer (GDSC) provides systematic drug-response measurements across cancer models [4]. These resources have enabled benchmark-scale modeling of pharmacogenomic interactions [5]. In a common regression setting, a DRP model receives cellular molecular profiles and drug structural information and predicts a continuous response value, such as log-transformed IC50.

Recent DRP models have improved predictive performance by enriching both cellular and drug representations. Multi-omics integration has been used to combine gene expression, mutation, DNA methylation, and other molecular profiles for cell-line representation, as in late-integration models such as MOLI [6]. Pathway-aware and broader multi-source models further incorporate pathway enrichment [7], pathway-difference features [8], or transformer-based multi-omics fusion [9]. On the drug side, convolutional models have encoded SMILES-derived drug features [10], graph-based models have represented compounds as molecular graphs [11], and transformer-based models have learned sequence-style drug representations [12]. Relational modeling has also been introduced through protein-association-based cell graphs [13], drug–cell pair graphs [14], heterogeneous cell-line–drug networks [15], and pathway-guided graph convolution [16]. These studies show that multi-source representation and relational modeling are useful for DRP, but many existing designs still use omics views mainly as parallel feature sources or perform cell-side relation learning after feature integration. This assumption can be limiting because two cell lines may be close in pathway activity but distant in mutation or DNA methylation space, suggesting that different omics profiles do not necessarily define the same cell-line neighborhood. This motivation is consistent with prior multi-omics methods that construct separate sample similarity networks or graphs for different omics data [17, 18]. In our analysis, the model assigned different view weights across drugs, and higher weights were associated with larger increases in drug-wise prediction error when the corresponding view was removed (Supplementary Fig. S3). Early feature fusion can compress such relations into a single cell-line representation, while a single integrated cell graph can obscure which omics view defines the cell-line relation used for propagation. This motivates a relation-level multi-view formulation that preserves omics-view-specific cell-line relation structures before drug-conditioned cell–drug interaction.

Here, we propose MulDR (Fig. 1), a structured multi-view relational learning framework for DRP. MulDR treats pathway activity, gene expression, mutation, and DNA methylation as distinct omics views, and constructs a view-specific hypergraph from each view to describe cell-line relations under that molecular context. These hypergraphs are encoded separately to learn omics-specific cell representations, which are then adaptively fused under the guidance of multi-granular drug representations derived from molecular motifs, three-dimensional molecular graphs, and Morgan fingerprints. In addition, MulDR uses a training-induced bipartite graph branch to obtain an auxiliary cell embedding from observed training cell–drug response pairs, and integrates it with the drug-conditioned multi-view cell representation through gated cell integration. This design allows each omics view to contribute its own cell-line relation pattern, while the drug representation determines how these views are combined for response prediction. We evaluate MulDR on a GDSC/CCLE drug response benchmark under a standard pair-level split and cold-start protocols. MulDR outperforms representative and recent baselines in the standard setting and maintains strong performance when predicting responses for unseen drugs or cell lines. Ablation analyses further show that omics-view-specific cell relation modeling, drug-conditioned view fusion, and training-induced graph augmentation are key contributors to the final performance. In summary, MulDR provides an effective multi-view framework for DRP by modeling omics-specific cell-line relations and coupling the learned cell representations with drug representations.

**Figure 1:**
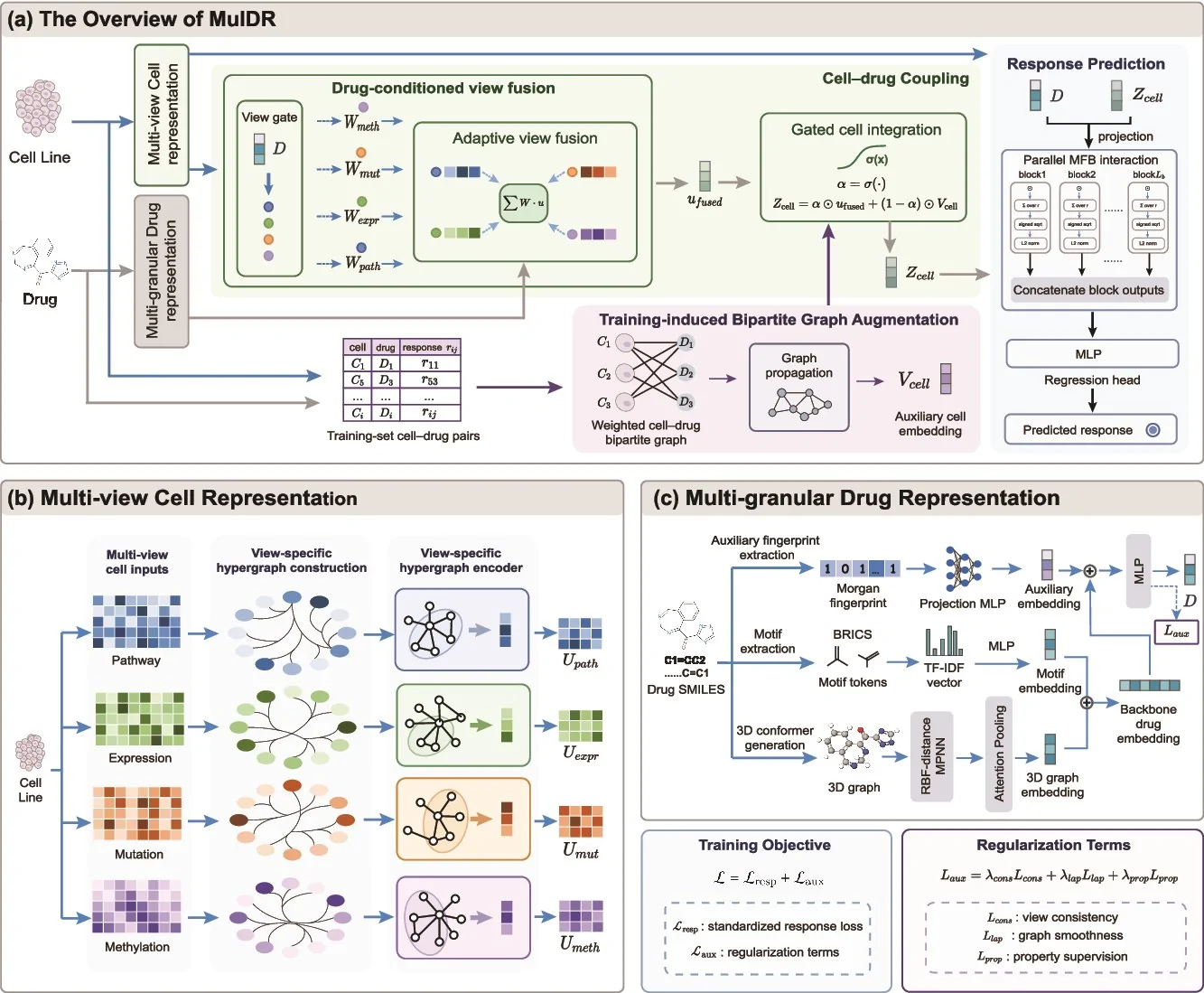
Overview of MulDR. (a) Overall framework of MulDR, including drug-conditioned view fusion, training-induced bipartite graph augmentation, gated cell integration, parallel MFB interaction blocks, and response prediction. (b) Multi-view cell representation based on view-specific hypergraph construction and encoding. (c) Multi-granular drug representation based on motif extraction, three-dimensional molecular graph modeling, and Morgan fingerprint extraction and projection.

## Related work

### Multi-source drug response prediction

Large-scale pharmacogenomic resources have enabled supervised models that connect cell-line molecular profiles with drug-response measurements. CCLE provides molecular and pharmacological characterization of cancer cell lines [3], and GDSC provides systematic drug-sensitivity measurements across cancer models [4]. Data from these resources have supported benchmark studies of drug sensitivity prediction [19] and analyses of pharmacogenomic interactions [5]. CTRPv2 [20], gCSI [21], and PRISM [22] have further broadened the available drug-response data. Together, these resources provide a foundation for DRP models that combine cellular molecular profiles with drug information.

Multi-omics DRP models use heterogeneous cellular profiles to characterize cancer cell states. MOLI integrates multiple omics views through late integration [6], while DeepCDR combines genomic features with a graph-based drug representation [23]. More recent models further incorporate pathway-level information or broader multi-source representations. BANDRP uses pathway enrichment scores together with multi-omics cell features and drug fingerprints [7], PASO constructs pathway-based multi-omics difference features [8], and MTEGDRP integrates multi-omics cell features with molecular self-attention and equivariant graph neural networks [9]. These studies show that richer cellular and chemical inputs can improve DRP, but their multi-omics components are mainly used as feature sources rather than as separate relation-defining spaces.

Drug representation learning has also expanded from handcrafted descriptors to learned molecular encoders. tCNNS uses convolutional modeling for drug-response prediction from drug-related input representations [10], GraphDRP represents drugs with graph convolutional networks [11], and DeepTTA uses a transformer-based architecture for cancer drug-response prediction [12]. Related molecular representation and drug-related prediction studies have further explored molecular substructure attention [24], multimodal molecular fusion [25], cross-modal information flow with hierarchical attention [26], structured semantic extraction for molecular representation learning [27], multimodal feature fusion for drug-target interaction prediction [28], and cross-modal feature fusion for anticancer drug synergy prediction [29]. These works provide methodological context for the complementary drug representations used in MulDR. In our framework, the learned drug representation provides the conditioning signal for fusing omics-view-specific cell representations.

### Relational modeling for drug response prediction

Relational inductive bias has been introduced into DRP to exploit dependencies among cells, drugs, or cell–drug pairs. Twin graph neural networks with similarity augmentation (TGSA) constructs protein-association-based cell graphs for drug-response prediction [13], whereas GADRP builds drug–cell pair graphs with graph convolution and autoencoding components [14]. GraphTCDR constructs a heterogeneous cell-line–drug network using multi-omics and drug features as node attributes [15]. GCNPath uses pathway-guided graph convolution and emphasizes benchmark evaluation and external validation [16]. Hypergraph contrastive learning and graph transformer for drug response prediction (HCLGT-DRP) further constructs omics-specific hypergraphs and combines them with graph-transformer-based drug representation for DRP [30]. These graph-based models show the utility of relational modeling, but cell-side relations are commonly represented or fused into a unified structure rather than preserved as separate omics-specific relation spaces for subsequent drug-conditioned interaction.

Hypergraph and multi-view graph learning provide useful methodological context for modeling higher-order and view-dependent relations. Hypergraph neural networks model non-pairwise relations through hyperedges [31], and hypergraph attention further improves representation learning on high-order graph-structured data [32]. Multi-view hypergraph learning has also been used to capture complementary relation structures across views [33], and hypergraph multi-view attention further supports relational representation learning from multiple views [34]. These methods support the general idea that different views can define complementary relation structures. MulDR adapts this principle to DRP by treating pathway activity, gene expression, mutation, and DNA methylation as separate relation-defining omics views and by delaying their fusion until the queried drug is available.

## Materials and methods

### Problem definition

Let $\mathcal{C}=\{c_{1},\ldots ,c_{N_{c}}\}$ denote the set of $N_{c}$ cell lines and $\mathcal{D}=\{d_{1},\ldots ,d_{N_{d}}\}$ denote the set of $N_{d}$ drugs. Each observed sample is a triplet $(c_{i},d_{j},y_{ij})$, where $y_{ij}\in \mathbb{R}$ is the GDSC2 LN_IC50 value of drug $d_{j}$ on cell line $c_{i}$. During training, the response is transformed into a standardized value $\tilde{y}_{ij}$ using statistics fitted only on the training partition. Given a cell–drug pair $(c_{i},d_{j})$, MulDR is trained to predict the corresponding standardized response $\hat{\tilde{y}}_{ij}$. The prediction on the original log-transformed IC50 scale, denoted by $\hat{y}_{ij}$, is obtained by inverse standardization during evaluation.

### Overview of MulDR

The overall architecture of MulDR is illustrated in Fig. 1a. MulDR consists of three main components: multi-view cell representation learning, multi-granular drug representation learning, and drug-conditioned cell–drug coupling for response prediction. On the cell side, pathway activity, gene expression, mutation, and DNA methylation are used as separate omics views. For each view, MulDR constructs a view-specific hypergraph and encodes it with a view-specific hypergraph encoder, producing omics-view-specific cell representations (Fig. 1b). On the drug side, MulDR extracts complementary molecular information from motif tokens, three-dimensional molecular graphs, and Morgan fingerprints, and integrates them into a multi-granular drug embedding (Fig. 1c).

During response prediction, the multi-granular drug embedding is used to generate drug-conditioned view weights for adaptive fusion of the omics-view-specific cell representations. In parallel, a training-induced bipartite graph augmentation branch constructs a weighted cell–drug graph from observed training-set cell–drug response pairs and produces an auxiliary cell embedding through graph propagation. The drug-conditioned fused cell representation and the auxiliary response-derived cell embedding are then integrated through a gated cell integration module. Finally, the integrated cell representation and drug embedding pass through parallel multimodal factorized bilinear pooling (MFB) blocks and a multilayer perceptron (MLP) regression head to predict the standardized drug response, which is transformed back to the log-transformed IC50 scale during evaluation.

### Multi-view cell representation learning

#### View-specific hypergraph construction

We selected 733 genes from the COSMIC Cancer Gene Census [35] and matched them to processed CCLE gene expression, mutation, and DNA methylation profiles [3]. This gave 714 expression features, 715 mutation features, and 603 methylation features. Multiple mutation sites in one gene were combined into a single binary indicator. Supplementary Table S1 summarizes the feature counts and selection steps. We obtained human pathway gene sets from MSigDB C2:CP [36]. After intersecting these sets with the 714 retained expression genes and removing sets with fewer than five overlapping genes, 1,283 pathways remained. We calculated pathway activity from gene expression using gene set variation analysis (GSVA) with a Gaussian kernel [37]. GSVA scores were calculated for the molecular cell-line collection before the drug-response split.

Different omics profiles can induce different cell-line neighborhood structures. A pair of cell lines may be close in pathway activity but distant in mutation or DNA methylation, and collapsing these views before relation learning may obscure such view-dependent cell-line relations. Therefore, MulDR uses four relation-defining omics views, denoted by $\mathcal{V}=\{v_{\mathrm{p}},v_{\mathrm{g}},v_{\mathrm{m}},v_{\mathrm{D}}\}$, corresponding to pathway activity, gene expression, mutation, and DNA methylation. For each view $v\in \mathcal{V}$, let $\mathbf{F}^{(v)}\in \mathbb{R}^{N_{c}\times p_{v}}$ denote the cell-line feature matrix, where $p_{v}$ is the number of features in view *v*. Pathway activity, gene expression, and DNA methylation are represented as continuous-valued views, whereas mutation is represented as a binary view. All four hypergraphs contain the same cell lines as nodes; their hyperedges and weights differ because they use different molecular profiles. For each split, we standardized pathway scores using training cells and applied the same values to validation and test cells. Each pathway defined one hyperedge. For pathway *p*, the threshold $t_{p}$ was the 50th-largest absolute standardized score among training cells. Cell line $c_{i}$ joined the pathway hyperedge when $|z_{ip}|\geq t_{p}$, where $z_{ip}$ is its standardized pathway score. Gene expression and DNA methylation neighborhoods were based on cosine similarity. For the mutation view, we used Tanimoto similarity. For two binary mutation profiles $\mathbf{x}_{i}^{(\mathrm{mut})}$ and $\mathbf{x}_{j}^{(\mathrm{mut})}$, similarity is defined as


(1) \begin{align*}& { s_{ij}^{(\mathrm{mut})} = \frac{(\mathbf{x}_{i}^{(\mathrm{mut})})^{\top}\mathbf{x}_{j}^{(\mathrm{mut})}} {\|\mathbf{x}_{i}^{(\mathrm{mut})}\|_{1}+\|\mathbf{x}_{j}^{(\mathrm{mut})}\|_{1}-(\mathbf{x}_{i}^{(\mathrm{mut})})^{\top}\mathbf{x}_{j}^{(\mathrm{mut})}}.}\end{align*}

For each of these three views, we represented the top-*K* neighbors of each cell line as a hyperedge. The four hypergraphs were constructed from the corresponding molecular profiles.

Let $\mathbf{M}^{(v)}$ denote the weighted incidence matrix of the hypergraph for view *v*. The corresponding normalized cell-line propagation operator is defined as


(2) \begin{align*}& \mathbf{S}^{(v)} = (\mathbf{D}^{(v)}_{n})^{-\frac{1}{2}} \mathbf{M}^{(v)} (\mathbf{D}^{(v)}_{e})^{-1} (\mathbf{M}^{(v)})^{\top} (\mathbf{D}^{(v)}_{n})^{-\frac{1}{2}},\end{align*}

where $\mathbf{S}^{(v)}\in \mathbb{R}^{N_{c}\times N_{c}}$ denotes the normalized cell-line propagation operator induced by the view-specific hypergraph, and $\mathbf{D}^{(v)}_{n}$ and $\mathbf{D}^{(v)}_{e}$ are the node-degree and hyperedge-degree matrices computed from the weighted incidence matrix $\mathbf{M}^{(v)}$, respectively.

#### View-specific relation propagation

After each view-specific hypergraph is converted into the normalized propagation operator $\mathbf{S}^{(v)}$, MulDR learns a relational cell representation by propagating information over the corresponding omics-induced cell-line relation structure. For each omics view *v*, the view-specific relational cell representation is defined as


(3) \begin{align*}& \mathbf{U}^{(v)} = \phi^{(v)}_{\mathrm{rel}}(\mathbf{F}^{(v)},\mathbf{S}^{(v)}) \in\mathbb{R}^{N_{c}\times d_{c}},\end{align*}

where $\phi ^{(v)}_{\mathrm{rel}}(\cdot )$ denotes the view-specific relation propagation module, $d_{c}$ is the cell embedding dimension, and the *i*th row $\mathbf{u}^{(v)}_{i}=\mathbf{U}^{(v)}_{i,:}$ denotes the embedding of cell line $c_{i}$ in view *v*.

The module first projects the original view-specific features into the hidden space:


(4) \begin{align*}& \mathbf{H}^{(0)} = \mathbf{F}^{(v)}\mathbf{W}^{(v)}_{\mathrm{in}},\end{align*}

where $\mathbf{W}^{(v)}_{\mathrm{in}}$ is a trainable input projection matrix, and the *i*th row $\mathbf{h}^{(0)}_{i}=\mathbf{H}^{(0)}_{i,:}$ denotes the initial hidden representation of cell line $c_{i}$.

Attention-based local aggregation is then performed on the nonzero support of the hypergraph-induced propagation operator. At attention layer *l*, let $\mathbf{h}^{(l)}_{i}$ denote the hidden representation of cell line $c_{i}$. It is projected as


(5) \begin{align*}& \mathbf{q}^{(l)}_{i} = \mathbf{W}^{(l)}\mathbf{h}^{(l)}_{i},\end{align*}

where $\mathbf{W}^{(l)}$ is a trainable projection matrix and $\mathbf{q}^{(l)}_{i}$ is the projected representation. For structurally supported neighbors with $\mathbf{S}^{(v)}_{ij}>0$, the attention coefficient is computed as


(6) \begin{align*}& \alpha_{ij}^{(l)} = \operatorname{softmax}_{j:\mathbf{S}^{(v)}_{ij}>0} \left( a^{(l)}(\mathbf{q}^{(l)}_{i},\mathbf{q}^{(l)}_{j}) \right),\end{align*}

where $a^{(l)}(\cdot ,\cdot )$ is a trainable attention scoring function. Within a view and attention layer, $\alpha _{ij}^{(l)}$ weights the contribution of neighbor cell $c_{j}$ to cell $c_{i}$. It is calculated only for pairs with $\mathbf{S}^{(v)}_{ij}>0$. The representation of cell line $c_{i}$ is updated by


(7) \begin{align*}& \mathbf{h}^{(l+1)}_{i} = \sigma_{\mathrm{act}} \left( \sum_{j:\mathbf{S}^{(v)}_{ij}>0} \alpha_{ij}^{(l)} \mathbf{S}^{(v)}_{ij} \mathbf{q}^{(l)}_{j} \right),\end{align*}

where $\sigma _{\mathrm{act}}(\cdot )$ is an activation function, $\alpha _{ij}^{(l)}$ learns adaptive neighbor importance, and $\mathbf{S}^{(v)}_{ij}$ retains the structural weight induced by hyperedge co-membership.

To capture longer-range cell-line dependencies beyond the immediate hypergraph-induced neighborhood, the attention output is further propagated using an update based on approximate personalized propagation of neural predictions (APPNP) [38]. Let $L_{\mathrm{att}}$ denote the number of attention layers, and let


(8) \begin{align*}& \mathbf{R}^{(0)} = \mathbf{H}^{(L_{\mathrm{att}})}\end{align*}

be the initial representation for APPNP propagation. The propagation step is defined as


(9) \begin{align*}& \mathbf{R}^{(t+1)} = (1-\rho)\mathbf{S}^{(v)}\mathbf{R}^{(t)} + \rho\mathbf{R}^{(0)}, \quad t=0,\ldots,T-1,\end{align*}

where *ρ * is the restart coefficient, and *T* is the number of propagation steps. The final view-specific cell representation is obtained by adding a skip projection from the original omics features:


(10) \begin{align*}& \mathbf{U}^{(v)} = \operatorname{LayerNorm} \left( \mathbf{R}^{(T)} + \mathbf{F}^{(v)}\mathbf{W}^{(v)}_{\mathrm{skip}} \right),\end{align*}

where $\mathbf{W}^{(v)}_{\mathrm{skip}}$ projects the original features of view *v* into the cell embedding space. The set $\{\mathbf{U}^{(v)}\}_{v\in \mathcal{V}}$ forms the structured multi-view cellular representation used for drug-conditioned fusion.

### Multi-granular drug representation learning

The queried drug provides the conditioning signal for view fusion and the molecular counterpart for cell–drug interaction modeling. Because different molecular descriptors capture different aspects of chemical structure, MulDR encodes each drug using complementary motif-level, geometry-aware, and Morgan fingerprint representations, and then fuses them into the final drug embedding $\mathbf{d}_{j}$.

#### Motif-level drug representation

Motif-level descriptors provide sparse substructural information by capturing recurring molecular fragments and functional groups. MulDR therefore constructs a training-set motif vocabulary $\mathcal{M}$ from BRICS fragments [39] and predefined functional-group SMARTS matches using RDKit [40]. Vocabulary filtering and inverse document frequency statistics are fitted only on training drugs and then fixed for validation and test drugs.

For drug $d_{j}$, let $\mathbf{c}_{j}\in \mathbb{R}^{|\mathcal{M}|}$ denote its motif count vector. The count vector is converted into a term frequency–inverse document frequency (TF–IDF) vector, where motif tokens are treated as terms and drugs are treated as documents. For motif *m*, the inverse document frequency is computed from the training drugs as


(11) \begin{align*}& \operatorname{idf}_{m} = \log \frac{1+N_{\mathrm{tr}}^{\mathrm{drug}}}{1+\operatorname{df}_{m}} + 1,\end{align*}

where $N_{\mathrm{tr}}^{\mathrm{drug}}$ is the number of training drugs and $\operatorname{df}_{m}$ is the number of training drugs containing motif *m*. The normalized motif vector is then computed as


(12) \begin{align*}& \mathbf{m}_{j} = \operatorname{L2Norm} \left( [c_{jm}\operatorname{idf}_{m}]_{m=1}^{|\mathcal{M}|} \right).\end{align*}

The motif embedding $\mathbf{e}^{\mathrm{motif}}_{j}$ is obtained by projecting the normalized TF–IDF motif vector through an MLP.

#### Geometry-aware molecular representation

While motif descriptors summarize substructural patterns, they do not explicitly encode three-dimensional molecular geometry. To incorporate geometry-aware chemical information, MulDR constructs a molecular graph with atom features and conformer-derived coordinates from the drug SMILES string. A three-dimensional conformer is generated using RDKit ETKDG [41] and then optimized with a force field using RDKit [40]. Each drug is represented by an atom feature matrix $\mathbf{X}_{j}$, a coordinate matrix $\mathbf{P}_{j}$, and a directed molecular graph.

For an edge *(p→ q)*, the inter-atomic distance is computed as


(13) \begin{align*}& r_{pq} = \|\mathbf{p}_{j,p}-\mathbf{p}_{j,q}\|_{2},\end{align*}

where $\mathbf{p}_{j,p}$ and $\mathbf{p}_{j,q}$ are the coordinate vectors of atoms *p* and *q* taken from $\mathbf{P}_{j}$. The distance is expanded by radial basis functions and passed through an edge network:


(14) \begin{align*}& \mathbf{g}_{pq} = \operatorname{MLP}_{e}(\psi(r_{pq})),\end{align*}

where $\psi (\cdot )$ denotes the radial basis expansion and $\mathbf{g}_{pq}$ modulates messages along edge *(p→ q)*. At layer *l*, let $\boldsymbol{\xi }^{(l)}_{p}$ denote the hidden representation of atom *p*. Atom *q* receives the distance-modulated message


(15) \begin{align*}& \boldsymbol{\eta}_{q}^{(l)} = \sum_{(p\rightarrow q)} \mathbf{g}_{pq}\odot\boldsymbol{\xi}_{p}^{(l)},\end{align*}

which is used in a residual update through the layer-specific network $\operatorname{MLP}_{\mathrm{atom}}^{(l)}$. Graph-level pooling then yields the geometry-aware drug embedding $\mathbf{e}^{\mathrm{3D}}_{j}$. This design follows molecular message-passing frameworks [42] and is related to continuous-filter molecular networks [43].

#### Drug fusion and auxiliary property supervision

The motif and 3D branches provide complementary drug information: the motif branch emphasizes sparse substructural patterns, whereas the geometry-aware branch captures atom-level spatial interactions. MulDR concatenates $\mathbf{e}^{\mathrm{motif}}_{j}$ and $\mathbf{e}^{\mathrm{3D}}_{j}$ and uses an MLP-based fusion mapping to obtain the backbone drug representation $\mathbf{d}^{(0)}_{j}$.

To provide a stable circular-substructure descriptor as the auxiliary fingerprint branch shown in Fig. 1c, MulDR computes a 2048-bit Morgan fingerprint from the drug SMILES string using RDKit [40, 44]. The fingerprint is projected by an MLP into a fingerprint embedding $\mathbf{e}^{\mathrm{fp}}_{j}$, which is then concatenated with $\mathbf{d}^{(0)}_{j}$ and fused by another MLP-based mapping to obtain the final drug embedding $\mathbf{d}_{j}$. During training, the backbone representation $\mathbf{d}^{(0)}_{j}$ is further regularized through an auxiliary molecular-property prediction head, which encourages the motif–geometry fusion branch to retain chemically informative descriptors before final fingerprint-level fusion.

### Drug-conditioned fusion and response prediction

#### Training-induced bipartite graph augmentation

The omics-view-specific cell representations are derived from molecular profiles, whereas observed training responses provide complementary information about cell–drug associations. MulDR therefore includes an auxiliary bipartite graph over all benchmark cell-line and drug nodes, $\mathcal{C}\cup \mathcal{D}$. Edges are added for observed training pairs. For each training pair $(c_{i},d_{j},y_{ij})$, the edge weight is derived from the corresponding standardized response. Held-out cell or drug nodes are retained in the graph without response edges.

Let $\mathbf{A}\in \mathbb{R}^{(N_{c}+N_{d})\times (N_{c}+N_{d})}$ denote the weighted adjacency matrix over $\mathcal{C}\cup \mathcal{D}$, with self-connections added, and let


(16) \begin{align*}& \mathbf{A}_{\mathrm{norm}} = \mathbf{D}_{A}^{-\frac{1}{2}} \mathbf{A} \mathbf{D}_{A}^{-\frac{1}{2}}\end{align*}

denote its normalized form, where $\mathbf{D}_{A}$ is the diagonal degree matrix of $\mathbf{A}$. A two-layer graph convolution is applied to $\mathbf{A}_{\mathrm{norm}}$ with node-type indicators as initial features, producing the auxiliary response-derived cell embedding matrix $\mathbf{B}\in \mathbb{R}^{N_{c}\times d_{c}}$ from the cell-line rows of the final layer. The *i*th row $\mathbf{b}_{i}=\mathbf{B}_{i,:}$ denotes the auxiliary response-derived embedding of cell line $c_{i}$.

#### Cell-drug coupling

**Drug-conditioned view fusion.** The view-specific cell representations preserve different omics-induced relation spaces, but their relevance can vary across queried drugs. To make the cell representation drug-aware before response prediction, MulDR uses the final drug embedding $\mathbf{d}_{j}$ to generate view-fusion weights over the omics-specific cell representations. Specifically,


(17) \begin{align*}& \mathbf{w}_{j} = \operatorname{softmax} \left( \frac{\operatorname{ViewGate}(\mathbf{d}_{j})}{\tau} \right) \in\mathbb{R}^{|\mathcal{V}|},\end{align*}

where $\operatorname{ViewGate}(\cdot )$ is an MLP that maps the drug embedding to view-level logits, *τ * is the temperature parameter, and $\mathbf{w}_{j}$ contains the fusion weights over omics views. The scalar $w_{j}^{(v)}$ denotes the weight assigned to view *v* for drug $d_{j}$. For pair $(c_{i},d_{j})$, the drug-conditioned multi-view cell representation is


(18) \begin{align*}& \mathbf{u}_{ij} = \sum_{v\in\mathcal{V}} w_{j}^{(v)}\mathbf{u}^{(v)}_{i}.\end{align*}

This operation allows the same cell line to use different mixtures of omics-view-specific relational representations when queried by different drugs. The four weights in $\mathbf{w}_{j}$ determine how the pathway, expression, mutation, and methylation representations are combined for drug $d_{j}$. The same weights are used for every cell line paired with that drug. They differ from the neighbor attention coefficients $\alpha _{ij}^{(l)}$.

**Gated cell integration.** The drug-conditioned multi-view representation $\mathbf{u}_{ij}$ and the response-derived auxiliary embedding $\mathbf{b}_{i}$ capture different cell-side signals. The former is derived from omics-specific cell-line relation spaces conditioned on the queried drug, whereas the latter summarizes training-response-derived cell–drug association patterns. To integrate these two sources adaptively, MulDR applies an element-wise gate:


(19) \begin{align*}& \boldsymbol{\beta}_{ij} = \operatorname{sigmoid} \left( \mathbf{W}_{u}\mathbf{u}_{ij} + \mathbf{W}_{b}\mathbf{b}_{i} \right),\end{align*}

where $\boldsymbol{\beta }_{ij}\in (0,1)^{d_{c}}$ is the integration gate, and $\mathbf{W}_{u}$ and $\mathbf{W}_{b}$ are trainable projection matrices. The gated cell integration module produces the final cell-side representation $Z_{\mathrm{cell}}$ shown in Fig. 1a, whose pair-specific form is computed as


(20) \begin{align*}& \mathbf{z}_{ij} = \boldsymbol{\beta}_{ij}\odot\mathbf{u}_{ij} + (\mathbf{1}-\boldsymbol{\beta}_{ij})\odot\mathbf{b}_{i},\end{align*}

where $\mathbf{1}$ is an all-one vector with the same dimension as $\boldsymbol{\beta }_{ij}$. The resulting $\mathbf{z}_{ij}$ is the cell-side representation used for cell–drug interaction modeling.

#### Factorized bilinear cell–drug interaction

After cell-side integration, the response of a pair $(c_{i},d_{j})$ depends on interactions between the final cell representation $\mathbf{z}_{ij}$ and the drug embedding $\mathbf{d}_{j}$. MulDR uses parallel MFB blocks to combine the cell and drug representations [45].

After layer normalization, $\mathbf{z}_{ij}$ and $\mathbf{d}_{j}$ are projected and reshaped into $\mathbf{P}_{z}^{(s)},\mathbf{P}_{d}^{(s)}\in \mathbb{R}^{K_{b}\times K_{f}}$ for each interaction block $s=1,\ldots ,L_{b}$, where $L_{b}$ is the number of parallel factorized blocks, $K_{b}$ is the block output dimension, and $K_{f}$ is the factorization rank. Let $\mathbf{p}_{z,k}^{(s)}$ and $\mathbf{p}_{d,k}^{(s)}$ denote the *k*th columns of $\mathbf{P}_{z}^{(s)}$ and $\mathbf{P}_{d}^{(s)}$, respectively. The factorized interaction output of block *s* is


(21) \begin{align*}& \mathbf{f}^{(s)} = \operatorname{Norm} \left( \sum_{k=1}^{K_{f}} \mathbf{p}_{z,k}^{(s)} \odot \mathbf{p}_{d,k}^{(s)} \right),\end{align*}

where $\operatorname{Norm}(\cdot )$ denotes the normalization operation in the interaction block. The independent block outputs are concatenated as


(22) \begin{align*}& \mathbf{f}_{ij} = [ \mathbf{f}^{(1)}; \ldots; \mathbf{f}^{(L_{b})} ],\end{align*}

and passed to an MLP regression head to predict the standardized response:


(23) \begin{align*}& \hat{\tilde{y}}_{ij} = \operatorname{MLP}_{\mathrm{pred}}(\mathbf{f}_{ij}).\end{align*}

The corresponding prediction on the log-transformed IC50 scale is obtained by inverse standardization during evaluation.

### Training objective

Following BANDRP [7], MulDR is trained on standardized drug-response values to reduce the influence of drug-dependent response scales. For an observed training pair $(c_{i},d_{j},y_{ij})$, where $y_{ij}$ denotes the log-transformed IC50 value, the standardized target is defined as


(24) \begin{align*}& \tilde{y}_{ij} = \frac{y_{ij}-\mu_{j}}{s_{j}+\epsilon}.\end{align*}

In the pair-level and cell-line cold-start settings, $\mu _{j}$ and $s_{j}$ were estimated only from the training responses for drug $d_{j}$. Specifically, $\mu _{j}$ is computed as a winsorized median and $s_{j}$ is computed from a median-absolute-deviation-based scale estimate. When the drug-specific estimate is unreliable because of too few training observations or a near-zero scale, it is stabilized using the corresponding global robust statistic estimated from the training partition. For drug cold start, one global center and scale were estimated from all training responses and used for validation and test drugs. The same values were used to transform predictions back to the log IC50 scale during evaluation.

For a mini-batch $\mathcal{B}$ of observed training pairs, the main response prediction loss is


(25) \begin{align*}& \mathcal{L}_{\mathrm{resp}} = \frac{1}{|\mathcal{B}|} \sum_{(c_{i},d_{j},y_{ij})\in\mathcal{B}} \left[ \left( \hat{\tilde{y}}_{ij} - \tilde{y}_{ij} \right)^{2} + \gamma_{\mathrm{h}} \ell_{\mathrm{SL1}} \left( \hat{\tilde{y}}_{ij}, \tilde{y}_{ij} \right) \right],\end{align*}

where $\hat{\tilde{y}}_{ij}$ is the predicted standardized response, $\ell _{\mathrm{SL1}}(\cdot )$ denotes the smooth L1 loss, and $\gamma _{\mathrm{h}}$ controls its contribution.

In addition to the main response loss, MulDR uses auxiliary regularization terms to stabilize representation learning. As shown in Fig. 1, the auxiliary objective consists of view-consistency regularization, graph-smoothness regularization, and molecular-property supervision:


(26) \begin{align*}& \mathcal{L}_{\mathrm{aux}} = \lambda_{\mathrm{cons}}\mathcal{L}_{\mathrm{cons}} + \lambda_{\mathrm{lap}}\mathcal{L}_{\mathrm{lap}} + \lambda_{\mathrm{prop}}\mathcal{L}_{\mathrm{prop}},\end{align*}

where $\lambda _{\mathrm{cons}}$, $\lambda _{\mathrm{lap}}$, and $\lambda _{\mathrm{prop}}$ control the contributions of the three auxiliary terms. These auxiliary losses are used only during training. Specifically, $\mathcal{L}_{\mathrm{cons}}$ is computed from omics-view-specific cell representations to improve cross-view compatibility, $\mathcal{L}_{\mathrm{lap}}$ regularizes cell representations over the training-induced cell relation structure, and $\mathcal{L}_{\mathrm{prop}}$ constrains the drug representation using RDKit-derived molecular descriptors computed from training-drug SMILES strings. The molecular-property targets include molecular weight, *łog P*, topological polar surface area, hydrogen-bond donors, hydrogen-bond acceptors, and ring count. These auxiliary objectives provide training-time constraints on the learned representations, while prediction is performed from the final cell–drug interaction representation.

The final objective is written as


(27) \begin{align*}& \mathcal{L} = \mathcal{L}_{\mathrm{resp}} + \mathcal{L}_{\mathrm{aux}},\end{align*}

where $\mathcal{L}_{\mathrm{aux}}$ denotes the weighted sum of auxiliary regularization terms, including view consistency, graph smoothness, and molecular-property supervision. During evaluation, standardized predictions are transformed back to the log-transformed IC50 scale.

## Experiments and results

### Experimental settings

**Datasets.** We evaluated MulDR on a benchmark assembled from CCLE molecular profiles [3] and GDSC v2 drug-response measurements [4]. The response matrix used the GDSC2 LN_IC50 field, which is the natural logarithm of fitted IC50 in *μ *M. The training targets were then standardized as described above [7], and evaluation errors were calculated after predictions were returned to the LN_IC50 scale. After removing missing response entries and aligning cell-line and drug identifiers, the benchmark contained 81 467 observed cell–drug pairs across 536 cell lines and 169 drugs. Drug structures were matched by PubChem identifiers and represented using valid SMILES strings from the drug information table [46].

For each cell line, four molecular views were used to characterize cellular states: pathway activity, gene expression, mutation, and DNA methylation. Before response alignment, the processed cellular feature matrices covered 823 cell lines. All 536 response-aligned cell lines were covered by the four views, yielding 1283 pathway activity features, 714 gene-expression features, 715 mutation features, and 603 DNA methylation features. Pathway activity, gene expression, and DNA methylation were treated as continuous-valued views, whereas mutation was represented as a binary view. Within each split, we calculated standardization values for the continuous omics features from training cells and used them unchanged for validation and test cells.

The main benchmark used a pair-level train/validation/test split with a ratio of 8:1:1, resulting in 65 175 training pairs, 8146 validation pairs, and 8146 test pairs. The validation set was used for model selection, and final performance was reported on the held-out test pairs. To assess entity-level generalization, we further used drug and cell-line cold-start protocols. In the drug cold-start protocol, drugs were split into 137 training drugs, 16 validation drugs, and 16 test drugs; the test drugs accounted for 9.47% of all drugs, and all corresponding test pairs involved drugs unseen during training. This split yielded 65 893 training pairs, 7963 validation pairs, and 7611 test pairs. In the cell-line cold-start protocol, cell lines were split into 430 training cell lines, 53 validation cell lines, and 53 test cell lines; the test cell lines accounted for 9.89% of all cell lines, and all corresponding test pairs involved cell lines unseen during training. This split yielded 65 340 training pairs, 8068 validation pairs, and 8059 test pairs. For each split, response centers and scales were calculated from training responses, feature standardization values and pathway thresholds from training cells, and motif vocabulary and IDF values from training drugs. The cell–drug response graph was built from training pairs. Pair-level and cell-line cold-start experiments used drug-specific response centers and scales, whereas drug cold-start used one global center and scale from all training responses. These values were also used to transform predictions back to log IC50 during evaluation.

**External evaluation settings.** We trained and selected the model on GDSC2 and evaluated it on CTRPv2 [20] and gCSI [21]. The drug evaluation used external compounds absent from GDSC2 and cell lines represented in the GDSC2 model. The cell-line evaluation used external cell lines with all four molecular profiles and drugs shared with GDSC2. We retained external responses with finite IC50 values and $r^{2}_{\mathrm{fit}}\geq 0.7$, and used the median when a cell–drug pair had multiple valid measurements. For each drug with at least 10 evaluable cell lines, we calculated Spearman and Pearson correlations across cell lines and then averaged across drugs [Macro-Spearman correlation coefficient (SCC) and Macro-Pearson correlation coefficient (PCC)]. Results are means *± * standard deviations over three random seeds. Cohort sizes and baseline results are in Supplementary Tables S3 and S4.

**Analysis of cell relations.** We compared the four omics views by measuring overlap among the 30 nearest neighbors of each cell line and cosine similarity between the off-diagonal entries of their propagation matrices. We assessed tissue association by comparing tissue labels among connected cells with 10 000 permutations of those labels, applying Holm correction across views. To test the contribution of each relation structure to prediction, we replaced one propagation matrix at a time with the identity matrix while retaining the original molecular features. Details are provided in Supplementary Methods S1 and Supplementary Table S2.

**Baselines and evaluation metrics.** We compared MulDR with seven representative and recent DRP baselines, including DeepCDR [23], GraphDRP [11], DeepTTA [12], BANDRP [7], PASO [8], GraphTCDR [15], and MTEGDRP [9]. These baselines cover hybrid graph convolution, molecular graph encoding, transformer-based drug representation, pathway-aware modeling, heterogeneous graph learning, geometry-aware molecular modeling, and multi-omics fusion. Performance is assessed using five metrics: root mean square error (RMSE), mean absolute error (MAE), coefficient of determination ($R^{2}$), PCC, and SCC. RMSE and MAE measure prediction errors, with lower values indicating better accuracy, whereas $R^{2}$, PCC, and SCC measure the agreement between observed and predicted responses, with higher values indicating better predictive performance. All baselines were retrained using the same train/validation/test splits and target standardization as MulDR. Predictions from all methods were returned to the LN_IC50 scale using training response statistics before the test metrics were calculated. MulDR and every baseline were run with five matched random seeds for Table 1 and Fig. 3. We used two-sided paired *t*-tests with Holm correction across seven baseline comparisons for each metric and evaluation setting.

**Table 1:** Performance comparison on the main benchmark test set.

Method	RMSE*↓ *	MAE*↓ *	$R^{2}\uparrow $	PCC*↑ *	SCC*↑ *
DeepCDR	1.0417 *± * 0.0183	0.8013 *± * 0.0127	0.8546 *± * 0.0061	0.9254 *± * 0.0042	0.9014 *± * 0.0076
GraphDRP	1.1073 *± * 0.0261	0.8489 *± * 0.0174	0.8358 *± * 0.0083	0.9137 *± * 0.0057	0.8890 *± * 0.0102
DeepTTA	1.0718 *± * 0.0217	0.8336 *± * 0.0149	0.8461 *± * 0.0054	0.9198 *± * 0.0036	0.8956 *± * 0.0089
BANDRP	0.9476 *± * 0.0094	0.6948 *± * 0.0068	0.8797 *± * 0.0032	0.9372 *± * 0.0021	0.9157 *± * 0.0047
PASO	0.9951 *± * 0.0156	0.7580 *± * 0.0103	0.8674 *± * 0.0046	0.9317 *± * 0.0038	0.9074 *± * 0.0063
GraphTCDR	0.9714 *± * 0.0129	0.7186 *± * 0.0081	0.8736 *± * 0.0027	0.9353 *± * 0.0019	0.9128 *± * 0.0051
MTEGDRP	1.1469 *± * 0.0314	0.8735 *± * 0.0196	0.8238 *± * 0.0097	0.9084 *± * 0.0064	0.8805 *± * 0.0118
MulDR	**0.8901 *± * 0.0071**	**0.6499 *± * 0.0053**	**0.8938 *± * 0.0018**	**0.9454 *± * 0.0013**	**0.9224 *± * 0.0034**

Results are reported as mean *± * standard deviation over five matched random-seed runs. Best results are shown in bold, and second-best results are underlined. Holm-adjusted *P* values for comparisons between MulDR and the baselines are reported in Supplementary Table S5.

### Predictive performance evaluation

**Main benchmark comparison.** To evaluate benchmark-level predictive accuracy, we compared MulDR with the selected DRP baselines under the matched pair split. As summarized in Table 1, MulDR achieved the best mean performance across all five metrics, with an RMSE of *0.8901± 0.0071* and a PCC of *0.9454± 0.0013*. Relative to the strongest baseline, MulDR reduced RMSE by 6.07%. The improvements over every baseline were significant for all five metrics after Holm correction ($P_{\mathrm{adj}}<0.05$; Supplementary Table S5).

The prediction-level diagnostics in Fig. 2 further examine the sample-level behavior of MulDR on the main test set. The observed-versus-predicted scatter plot in Fig. 2a shows that most predictions lie close to the diagonal, indicating good agreement across the response range. The residual distribution in Fig. 2b is centered near zero, suggesting no clear overall overestimation or underestimation. The residual-versus-fitted plot in Fig. 2c shows no evident monotonic pattern, indicating that prediction errors are not systematically associated with the fitted response level.

**Figure 2:**
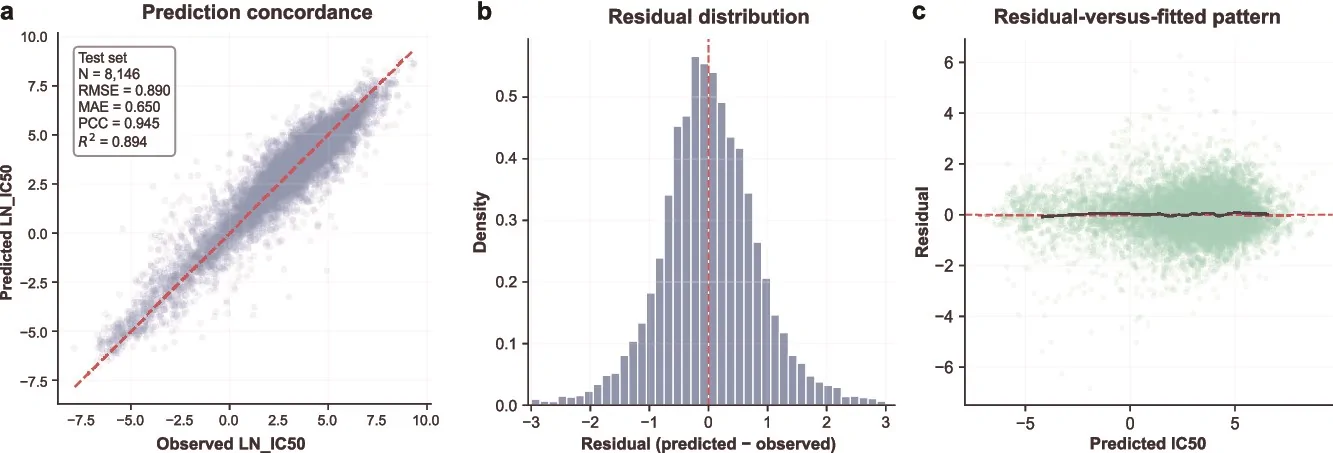
Prediction-level diagnostics on the main benchmark test set. (a) Prediction concordance between observed and predicted log-transformed IC50 values. (b) Distribution of residuals, defined as predicted minus observed values. (c) Residual-versus-fitted pattern across predicted response values.

**Figure 3:**
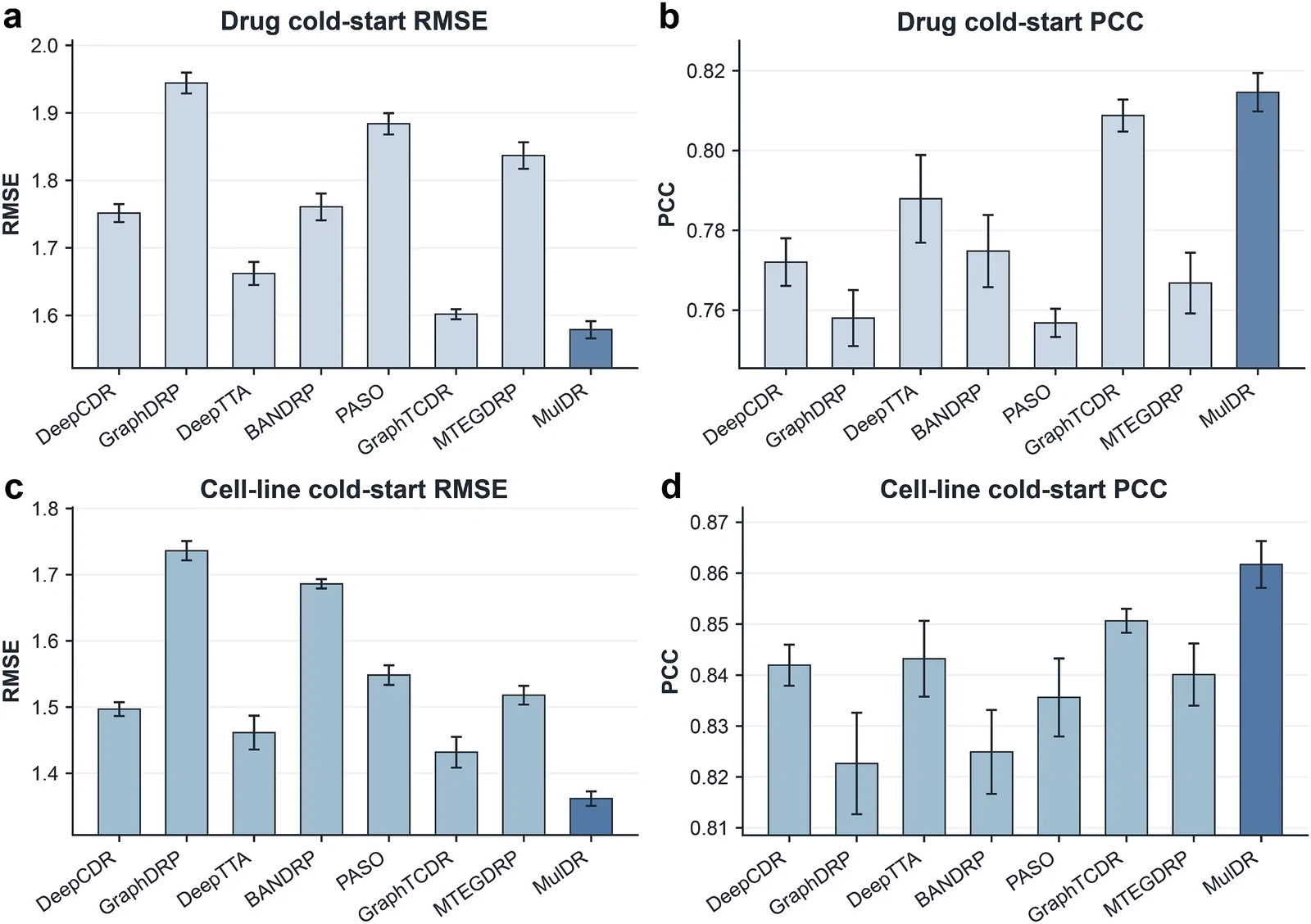
Drug and cell-line cold-start performance. (a) RMSE under the drug cold-start protocol. (b) PCC under the drug cold-start protocol. (c) RMSE under the cell-line cold-start protocol. (d) PCC under the cell-line cold-start protocol. Bars indicate mean performance over five matched random-seed runs, and error bars indicate standard deviation.

**Drug and cell-line cold-start evaluation.** We next evaluated entity-level generalization under the drug and cell-line cold-start protocols defined in the experimental settings. As shown in Fig. 3, MulDR achieved the strongest overall performance in both drug and cell-line cold-start settings, obtaining the lowest RMSE and the highest PCC among all compared methods. For cell-line cold-start, both metrics were significantly better than all baselines after Holm correction. For drug cold-start, MulDR achieved the lowest mean RMSE and the highest mean PCC. The RMSE improvements were significant against all baselines, and the PCC improvements were significant against six of the seven baselines after Holm correction (Supplementary Table S6). These results indicate that combining omics-view-specific cell relation modeling with multi-granular drug representation improves generalization to unseen drugs and cell lines.

**External evaluation.** MulDR had the highest mean Macro-SCC and Macro-PCC among the compared methods in all four external evaluations (Supplementary Table S4). On CTRPv2, Macro-SCC was *0.4936± 0.0061* for 384 external drugs and *0.4889± 0.0057* for 224 external cell lines. On gCSI, the corresponding values were *0.5174± 0.0068* for four external drugs and *0.5286± 0.0052* for 46 external cell lines. The CTRPv2 drug evaluation included 89 716 response pairs for compounds absent from GDSC2.

**Subgroup performance analysis.** To further examine whether the predictive behavior of MulDR was consistent across different biological contexts, we performed subgroup analysis on the main benchmark test set. Test samples were stratified according to the tissue annotation of the cell line and the pathway category of the queried drug, and RMSE and PCC were computed within each subgroup. As shown in Fig. 4, MulDR achieved lower RMSE and higher PCC than BANDRP across the displayed tissue and drug-pathway subgroups, indicating more consistent subgroup-level performance. MulDR maintained high PCC values across major tissue groups, whereas RMSE varied among tissues. The central nervous system subgroup showed relatively low prediction error, while large intestine, stomach, breast, and pancreas subgroups showed higher errors. The pathway-stratified results showed more pronounced variation across drug categories. Drugs associated with genome integrity and ERK MAPK signaling showed relatively low RMSE, whereas drugs in the DNA replication and apoptosis regulation categories showed higher prediction errors.

**Figure 4:**
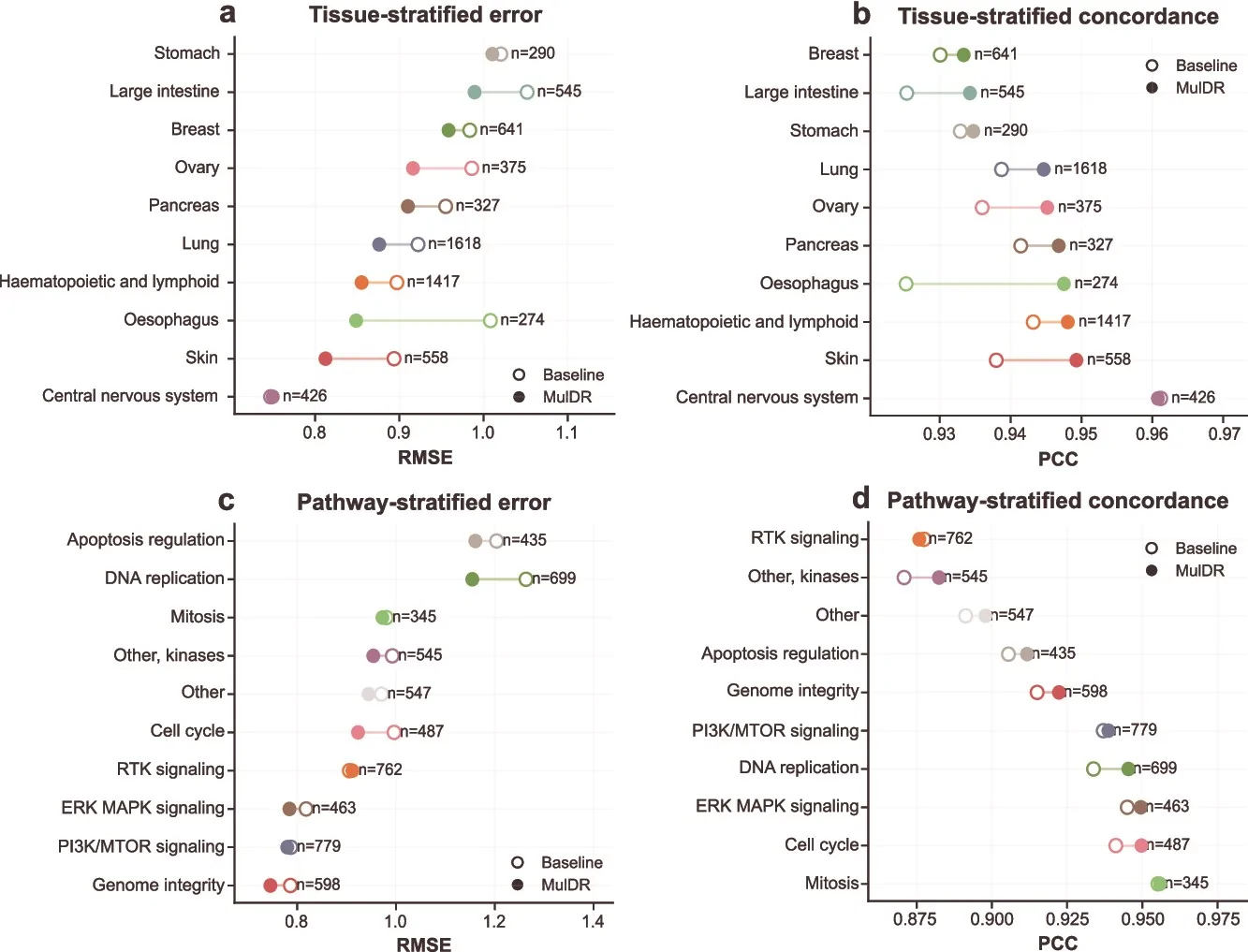
Subgroup performance analysis on the main benchmark test set. (a) Tissue-stratified RMSE. (b) Tissue-stratified PCC. (c) Drug-pathway-stratified RMSE. (d) Drug-pathway-stratified PCC. Text annotations indicate the number of test samples in each subgroup.

### Ablation analysis

To assess the contribution of each model component, we constructed controlled variants of MulDR in six groups.

- **Main variants. MulDR_noRg** removes the training-induced bipartite graph augmentation branch from MulDR.
- **Cell relation variants. MulDR_F** replaces omics-view-specific relational modeling with flat multi-omics feature integration; **MulDR_S** replaces separate omics-view-specific cell relation structures with a single integrated cell graph; **MulDR_noRP** removes relation propagation from the view-specific cell encoder; and **MulDR_noCR** removes cell-line relation modeling from the cell representation module.
- **View fusion variants. MulDR_G** replaces drug-conditioned view fusion with drug-independent global view fusion; **MulDR_Fi** uses fixed view-fusion weights; and **MulDR_D** uses a drug-shuffled view gate to disrupt the correspondence between queried drugs and view-fusion weights.
- **Response transformation variants. MulDR_Gs** replaces drug-specific robust response standardization with global response standardization, and **MulDR_R** directly predicts raw log-transformed IC50 values without the default response transformation.
- **Propagation removed from one view. MulDR_Expr-*I***, **MulDR_Mut-*I***, and **MulDR_Meth-*I*** replace the propagation matrix for the corresponding omics view with the identity matrix while retaining its molecular features.
- **Drug representations. MulDR_noMo**, **MulDR_noGe**, and **MulDR_noFp** remove the motif, 3D molecular graph, and Morgan fingerprint representations, respectively. **MulDR_Mo**, **MulDR_Ge**, and **MulDR_Fp** use only the corresponding representation.

Table 2 summarizes the ablation results on the main benchmark test set. Removing the training-induced bipartite graph augmentation branch led to a clear performance decrease, with RMSE increasing from 0.8901 to 0.9099, indicating that the auxiliary cell-line representation derived from observed cell–drug response associations provides complementary predictive information. Cell relation variants produced more substantial performance degradation. Among them, the flat multi-omics variant MulDR_F showed the largest decline, suggesting that preserving omics-view-specific cell-line relations before relation encoding is important for MulDR. MulDR_S, MulDR_noRP, and MulDR_noCR also decreased performance across all five metrics, further supporting the contribution of structured cell relation modeling. View fusion variants further verified the role of drug-conditioned omics integration. Compared with the full model, MulDR_G, MulDR_Fi, and MulDR_D all showed higher prediction errors and lower correlation metrics, indicating that the queried drug provides useful information for adaptively weighting different omics-view-specific cell representations. In addition, response transformation variants also reduced performance, especially MulDR_R, suggesting that the adopted response standardization strategy improves regression stability. Replacing propagation with the identity matrix in the expression, mutation, or methylation view increased RMSE while retaining the corresponding molecular features. Each two-representation drug model outperformed its constituent single-representation models across the five metrics. Among the two-representation models, MulDR_noMo had the lowest mean RMSE, while MulDR_noFp had the lowest mean MAE and highest mean SCC. The full model had the best mean value for each metric among these drug representation variants. Overall, these results show that omics-view-specific cell relation modeling, drug-conditioned view fusion, response-derived cell-line representation enhancement, response transformation, and complementary drug representations all contribute to the predictive performance of MulDR.

**Table 2:** Ablation study on the main benchmark test set.

Setting	RMSE*↓ *	MAE*↓ *	$R^{2}\uparrow $	PCC*↑ *	SCC*↑ *
*Main variants*					
MulDR	0.8901 *± * 0.0071	0.6499 *± * 0.0053	0.8938 *± * 0.0018	0.9454 *± * 0.0013	0.9224 *± * 0.0034
MulDR_noRg	0.9099 *± * 0.0295	0.6849 *± * 0.0076	0.8891 *± * 0.0078	0.9410 *± * 0.0049	0.9176 *± * 0.0034
*Cell relation variants*					
MulDR_F	0.9968 *± * 0.0334	0.7325 *± * 0.0145	0.8669 *± * 0.0019	0.9312 *± * 0.0055	0.9065 *± * 0.0040
MulDR_S	0.9764 *± * 0.0207	0.7169 *± * 0.0224	0.8723 *± * 0.0093	0.9349 *± * 0.0028	0.9129 *± * 0.0106
MulDR_noRP	0.9648 *± * 0.0080	0.7060 *± * 0.0234	0.8753 *± * 0.0030	0.9386 *± * 0.0009	0.9158 *± * 0.0047
MulDR_noCR	0.9480 *± * 0.0183	0.7021 *± * 0.0101	0.8796 *± * 0.0021	0.9404 *± * 0.0044	0.9152 *± * 0.0098
*View fusion variants*					
MulDR_G	0.9237 *± * 0.0178	0.6762 *± * 0.0077	0.8857 *± * 0.0025	0.9431 *± * 0.0065	0.9204 *± * 0.0067
MulDR_Fi	0.9346 *± * 0.0199	0.6855 *± * 0.0123	0.8830 *± * 0.0089	0.9393 *± * 0.0016	0.9188 *± * 0.0046
MulDR_D	0.9378 *± * 0.0158	0.6886 *± * 0.0170	0.8822 *± * 0.0042	0.9397 *± * 0.0060	0.9174 *± * 0.0022
*Response transformation variants*					
MulDR_Gs	0.9156 *± * 0.0160	0.6787 *± * 0.0096	0.8877 *± * 0.0094	0.9443 *± * 0.0017	0.9197 *± * 0.0125
MulDR_R	0.9433 *± * 0.0236	0.7015 *± * 0.0091	0.8808 *± * 0.0075	0.9416 *± * 0.0051	0.9180 *± * 0.0039
*Propagation removed from individual views*					
MulDR_Expr-*I*	0.9551 *± * 0.0301	0.7059 *± * 0.0191	0.8778 *± * 0.0048	0.9389 *± * 0.0011	0.9161 *± * 0.0072
MulDR_Mut-*I*	0.9314 *± * 0.0200	0.6885 *± * 0.0198	0.8838 *± * 0.0038	0.9423 *± * 0.0050	0.9185 *± * 0.0116
MulDR_Meth-*I*	0.9381 *± * 0.0084	0.7001 *± * 0.0062	0.8821 *± * 0.0015	0.9408 *± * 0.0064	0.9169 *± * 0.0036
*Drug representation variants*					
MulDR_noMo	0.9057 *± * 0.0165	0.6703 *± * 0.0069	0.8901 *± * 0.0047	0.9435 *± * 0.0026	0.9194 *± * 0.0031
MulDR_noFp	0.9088 *± * 0.0213	0.6637 *± * 0.0067	0.8894 *± * 0.0054	0.9430 *± * 0.0041	0.9197 *± * 0.0036
MulDR_noGe	0.9156 *± * 0.0108	0.6698 *± * 0.0135	0.8877 *± * 0.0027	0.9424 *± * 0.0019	0.9188 *± * 0.0062
MulDR_Ge	0.9353 *± * 0.0261	0.6836 *± * 0.0114	0.8828 *± * 0.0073	0.9396 *± * 0.0052	0.9158 *± * 0.0047
MulDR_Fp	0.9394 *± * 0.0176	0.6974 *± * 0.0159	0.8818 *± * 0.0043	0.9390 *± * 0.0035	0.9140 *± * 0.0081
MulDR_Mo	0.9638 *± * 0.0238	0.7027 *± * 0.0086	0.8756 *± * 0.0065	0.9358 *± * 0.0046	0.9116 *± * 0.0074

Results are means *± * standard deviations over five random-seed runs. The variants assess cell relations, view weighting, target standardization, and drug representations. Lower values indicate better performance for RMSE and MAE, whereas higher values indicate better performance for $R^{2}$, PCC, and SCC.

### Cell relations across omics views

All four views contain the same 536 cell lines, and none has an isolated node (Supplementary Table S2). Mean Jaccard overlap among the top-30 neighbors ranged from 0.0387 to 0.2454, while cosine similarity between propagation matrices ranged from 0.1309 to 0.4554 (Fig. 6(a)). Thus, the views connect the same cells in different ways. Tissue labels were used for the cell relation analysis. Connected cells shared tissue labels more often than expected after 10 000 label permutations in all four views (Holm-adjusted empirical $P=4.0\times 10^{-4}$ for each view; Fig. 6(b)). The tissue homophily scores were 0.3228 for expression and 0.2422 for DNA methylation. Replacing propagation with the identity matrix in one view at a time increased RMSE while keeping its molecular features (Table 2, Fig. 6c). These results support modeling cell relations separately for the four omics views.

**Figure 6:**
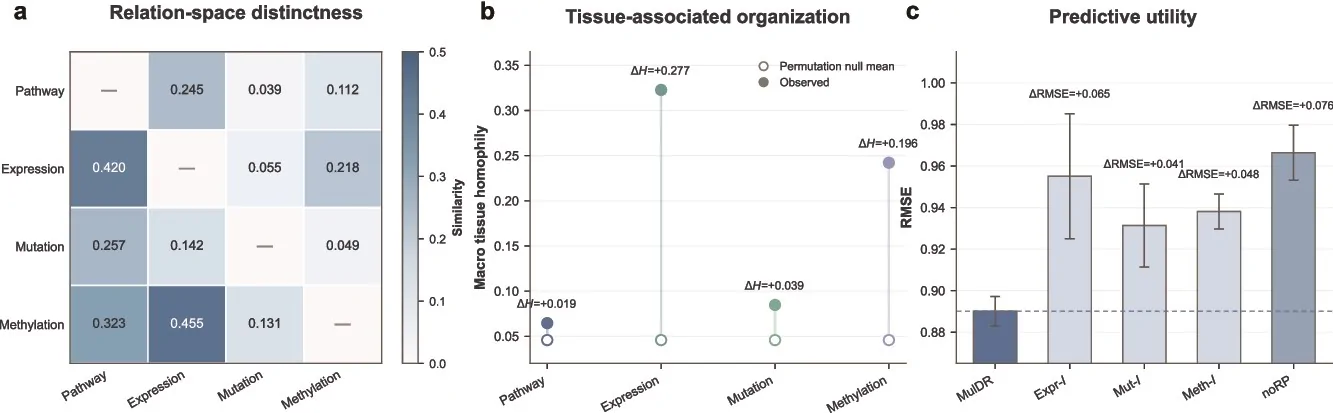
Cell relations across the four omics views. (a) Overlap among the 30 nearest cell lines in each view and similarity between propagation matrices. (b) Tissue label agreement in the observed graphs and after random label permutations. (c) RMSE after replacing propagation with the identity matrix in one view at a time; error bars show standard deviations over repeated runs.

### Parameter sensitivity analysis

We analyzed the number of attention heads, learning rate, KNN neighborhood size, and APPNP propagation steps. MulDR remained stable across the evaluated attention-head settings, and performance varied only slightly over the tested KNN sizes and APPNP depths (Supplementary Fig. S1). The final model used 8 attention heads, a learning rate of $1\times 10^{-3}$, *K=30* nearest neighbors, and *T=5* APPNP propagation steps based on validation performance.

### Representation analysis and case study

To examine whether MulDR learned structured representations beyond the original input space, we visualized raw features, BANDRP representations, and MulDR representations using UMAP. As shown in Fig. 5, raw features showed substantial overlap among drug pathway categories and tissue groups, while BANDRP representations exhibited more visible but still mixed local structures. In contrast, MulDR produced clearer pathway-level organization in the drug representation space and more compact tissue-related organization in the cell-line representation space, suggesting that MulDR learns more structured representations through multi-granular drug modeling and omics-view-specific cell relation learning.

**Figure 5:**
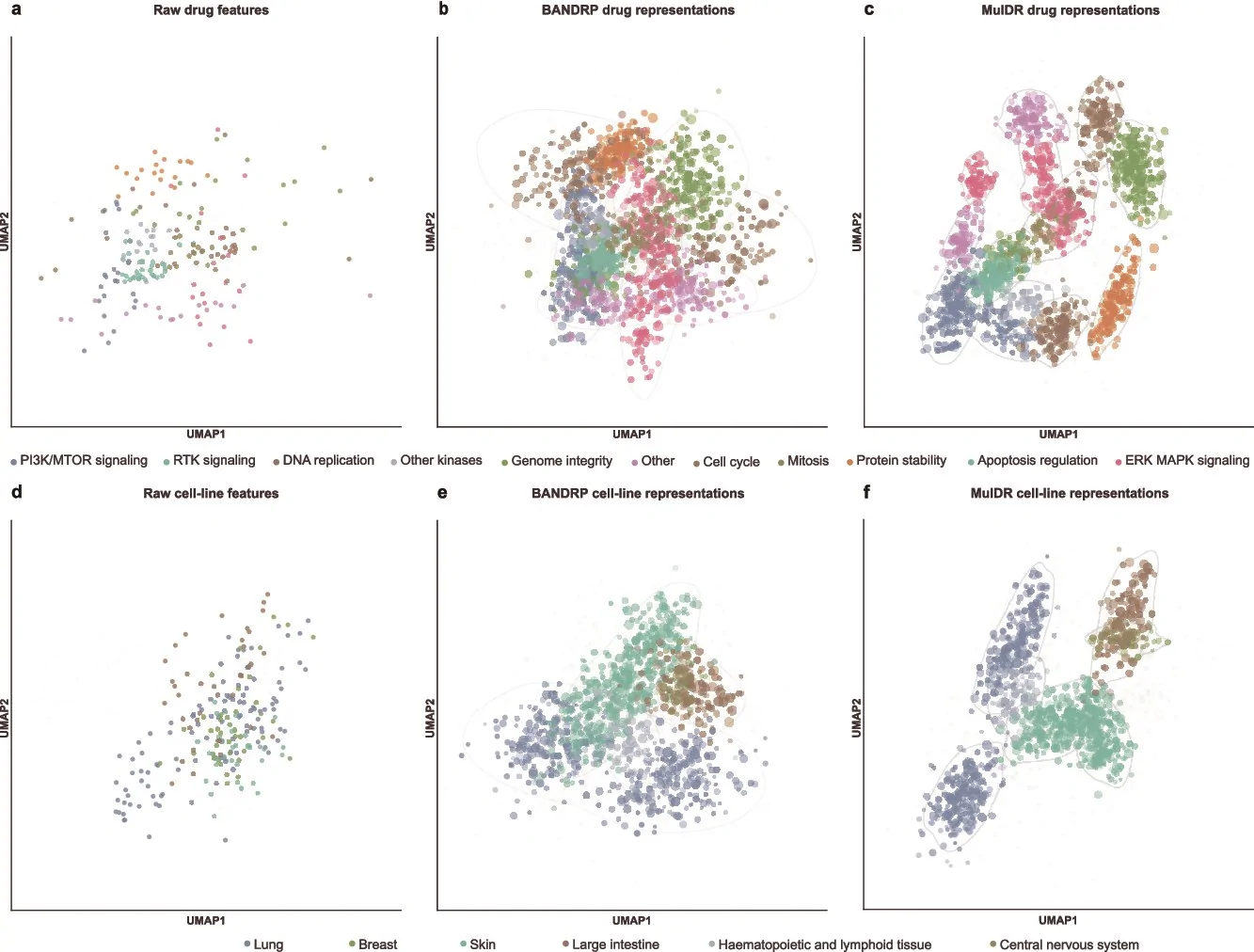
UMAP visualization of raw features, BANDRP representations, and MulDR representations. (a)–(c) show the drug feature and representation spaces colored by drug pathway category. (d)–(f) show the cell-line feature and representation spaces colored by tissue type.

We compared the motif and Morgan representations for all 169 benchmark drugs and 14,196 drug pairs (Supplementary Fig. S2). Drug-pair similarities were moderately correlated (Spearman *ρ =0.434*, 95% CI *[0.394,0.493]*, permutation $P<10^{-4}$). Mean Jaccard overlap among the nearest drugs was *0.163*, *0.184*, and *0.216* for *k=5*, 10, and 20. The learned representations also showed partial agreement (linear CKA *=0.635*; pairwise RSA *ρ =0.495*). Together with the ablation results in Table 2, these results show that the motif and Morgan representations provide complementary information.

The weights assigned to the four omics views differed across the 169 drugs (Supplementary Fig. S3). Mutation had the largest weight for 145 drugs, while pathway, expression, and methylation had the largest weight for 6, 8, and 10 drugs, respectively. Across drugs, a higher weight for a view was associated with a larger increase in drug-wise RMSE when that view was removed during prediction: Spearman *ρ =0.405* for pathway, *0.404* for expression, *0.224* for mutation, and *0.407* for methylation.

We selected PD0325901 for a single-drug case analysis because it had sufficient test samples across multiple tissue groups. As shown in Fig. 7, cell lines were ordered by observed log-transformed IC50 values to compare the predicted and observed response profiles. MulDR closely reproduced the overall response trend, with a drug-level RMSE of 0.563 and a PCC of 0.972.

**Figure 7:**
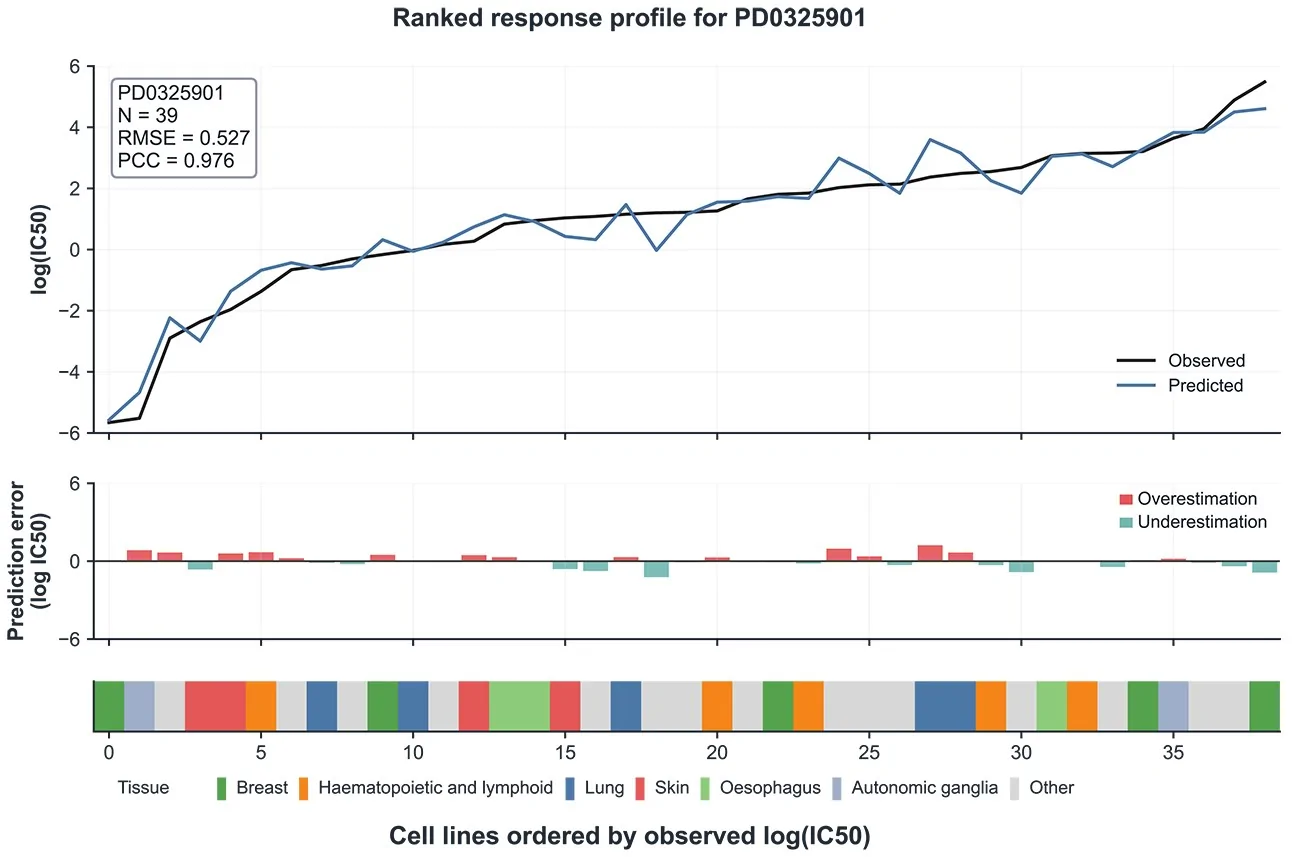
Single-drug response profile for PD0325901 on the main benchmark test set. Cell lines are ordered by observed log-transformed IC50 values. The response panel compares observed and predicted responses. The residual panel shows prediction residuals, with red bars indicating overestimation and green bars indicating underestimation. The annotation bar indicates coarse tissue groups.

## Discussion and Conclusion

We presented MulDR, a structured multi-view relational learning framework for DRP. MulDR models pathway activity, gene expression, mutation, and DNA methylation as omics-view-specific cell-line relation structures and adaptively fuses the resulting relational cell representations according to the queried drug. By further combining multi-granular drug representations and a training-response-derived auxiliary cell-line embedding, MulDR provides a relation-preserving predictive framework for heterogeneous cell–drug response modeling. Experiments on the GDSC/CCLE benchmark, together with cold-start evaluation, subgroup analysis, ablation studies, and representation analysis, validate the effectiveness and stability of the proposed framework. On CTRPv2 and gCSI, MulDR had the highest mean within-drug correlations among the compared methods in the four external evaluations.

Although MulDR shows promising performance, several aspects remain to be further investigated. While the CTRPv2 and gCSI evaluations extend beyond the GDSC/CCLE benchmark, further studies should examine generalization across additional cohorts, cancer types, and experimental settings. In addition, the biological meaning of the learned omics-view-specific cell-line relations requires deeper interpretation and analysis. Despite these limitations, the results indicate that preserving omics-specific cell-line relations is valuable for multi-omics drug response modeling and provides a structured modeling strategy for improving DRP in heterogeneous cancer contexts.

**Key Points**

- MulDR preserves omics-specific cell-line relation structures by modeling pathway activity, gene expression, mutation, and DNA methylation as separate relational views.
- MulDR uses drug-conditioned fusion to adaptively integrate multi-view cellular representations with multi-granular molecular drug representations.
- MulDR integrates molecular motifs, three-dimensional molecular graphs, and training-induced cell–drug associations to capture complementary chemical and response-derived information.
- MulDR improves prediction accuracy over representative baselines under standard and cold-start evaluation settings.

## Supplementary Material

MulDR_supplement_bbag619

## Data Availability

Codes and datasets of MulDR are available at https://github.com/Wang-ZengWei/MulDR.
