# Supplementary material for: Structured multi-view relational learning for drug response prediction
Source: Brief Bioinform. 2026 Nov 7;27(6):bbag619. doi: 10.1093/bib/bbag619 (PMC13744294; doi:10.1093/bib/bbag619)
Supplement: Supplementary file 1 [file muldr_supplement_bbag619.pdf]

Supplementary Material

Structured Multi-view Relational Learning for Drug Response Prediction

Supplementary Methods S1 Relation structure validation

For each cell line and each omics view, the top-30 non-self neighbors were selected according to the corresponding propagation operator $\mathbf{S}^{(v)}$. Pairwise neighborhood overlap was measured using the Jaccard index, while global propagation-structure similarity was measured using cosine similarity between the off-diagonal upper-triangular entries of the corresponding propagation matrices.

Tissue-associated organization was evaluated using cell-line tissue annotations that were not used for hypergraph construction or model training. All 536 benchmark cell lines were uniquely matched to tissue annotations. After removing three singleton tissue categories for which no within-tissue pair could be defined after excluding self-connections, the analysis included 533 cell lines from 21 tissue categories. For cell line i in view v, weighted tissue homophily was defined as

$$h_i^{(v)} = \frac{\sum_{j \neq i} S_{ij}^{(v)} \mathbb{I}(t_i = t_j)}{\sum_{j \neq i} S_{ij}^{(v)}}. \tag{1}$$

Homophily was first averaged within each tissue and then averaged equally across tissues. Enrichment was assessed using 10,000 tissue-label permutations while keeping the propagation matrices fixed, and empirical P values were corrected across the four views using the Holm procedure.

Predictive utility was evaluated using view-specific identity interventions. For expression, mutation, or DNA methylation, the corresponding propagation operator was set to the identity matrix while retaining the original molecular features and all remaining model components. Each intervention therefore removed cross-cell propagation in one view without removing its molecular feature content.

Supplementary Methods S2 Motif and Morgan representation analysis

The analysis included all 169 benchmark drugs and all 14,196 unique drug pairs. Descriptor-level correspondence was quantified by Spearman correlation between motif-vector cosine similarity and Morgan-fingerprint Tanimoto similarity. A drug-level bootstrap with 10,000 resamples was used to obtain the 95% confidence interval, and significance was assessed by 10,000 drug-label permutations. Local agreement was quantified as the Jaccard overlap between the top-k nearest-drug sets for $k = 5$, 10, and 20. Learned motif-branch and Morgan-branch embeddings were compared using linear centered kernel alignment and pairwise representational similarity analysis.

Supplementary Methods S3 Drug-conditioned view dependence

For each of the 169 drugs, the four normalized view-gate weights were extracted from the trained model. Model dependence on a view was measured by setting that view's relational representation to zero at inference time and calculating the resulting change in drug-wise RMSE. Spearman correlation across drugs was used to compare the gate weight of each view with its occlusion-induced RMSE change.

Supplementary Methods S4 Computational efficiency and scalability

MulDR contains 21.41 million trainable parameters and was trained on a single NVIDIA GeForce RTX 3090 GPU with a batch size of 128. Training ended after 19 epochs, with the best validation-RMSE checkpoint at epoch 17. The mean epoch wall-clock time, including training and validation, was 14.59 minutes; the training loop required 4 hours 37 minutes, and the end-to-end run required 4 hours 40 minutes. Peak allocated GPU memory was 4.75 GB. Inference on 8,146 test pairs required 95.39 seconds, corresponding to 11.71 ms per pair. Full hardware and software details are reported in Supplementary Table S7.

The relation matrices for the four omics views were constructed once and reused during training. For a new drug, MulDR computes motif, 3D molecular graph, and Morgan representations from its structure. In the cell-line cold-start and external cell-line evaluations, training cells determined the feature scaling values and pathway thresholds, and the other three views used hyperedges centered on training cells. Evaluated cell lines were connected using their molecular profiles.

Supplementary Table S1 Cellular feature definitions

View	Candidate space	Definition and filtering	Dimension
Gene expression	733 COSMIC CGC genes	Retain candidate genes represented in the processed expression matrix.	714
Mutation	733 COSMIC CGC genes	Merge multiple mutation sites at gene level and encode gene-level mutation status as binary.	715
DNA methylation	733 COSMIC CGC genes	Retain candidate genes represented in the processed methylation matrix.	603
Pathway activity	MSigDB C2:CP pathways	Intersect with 714 expression genes, retain pathways with at least five overlapping genes, and calculate GSVA scores with a Gaussian kernel.	1,283

Supplementary Table S2 Hypergraph structural audit

View	Nodes	Hyperedges	Mean edge size	Mean degree	Min degree	Isolated nodes
Pathway	536	1,283	50.0	119.68	25	0
Expression	536	536	30.0	30.00	4	0
Mutation	536	536	30.0	30.00	2	0
DNA methylation	536	536	30.0	30.00	3	0

Hyperedge size and node degree are computed from the nonzero support of the incidence matrix before propagation normalization. All four hypergraphs use the same 536 cell-line nodes, and every cell participates in at least one hyperedge in every view.

Supplementary Table S3 External evaluation cohorts

Dataset	Task	Candidate drugs	Candidate cells	Candidate pairs	Evaluated drugs	Evaluated cells	Evaluated pairs	Excluded drugs	Excluded pairs
CTRPv2	Drug generalization	416	486	89,873	384	486	89,716	32	157
CTRPv2	Cell-line generalization	76	224	9,301	74	224	9,292	2	9
gCSI	Drug generalization	4	258	558	4	258	558	0	0
gCSI	Cell-line generalization	12	46	348	10	46	341	2	7

For drug generalization, the cell count denotes evaluation cell lines represented in the GDSC2 model context. For cell-line generalization, it denotes external cell lines absent from the GDSC2 model-cell universe. Only drugs with at least 10 evaluable cell lines were included in the macro-averaged metrics.

Supplementary Table S4 External cross study performance

Method	CTRPv2 Drug		CTRPv2 Cell-line		gCSI Drug		gCSI Cell-line	
	SCC	PCC	SCC	PCC	SCC	PCC	SCC	PCC
DeepCDR	0.4526 ± 0.0147	0.4371 ± 0.0103	0.3814 ± 0.0283	0.3667 ± 0.0225	0.4057 ± 0.0249	0.3829 ± 0.0202	0.3728 ± 0.0334	0.3516 ± 0.0279
GraphDRP	0.4187 ± 0.0216	0.4049 ± 0.0168	0.4569 ± 0.0114	0.4398 ± 0.0078	0.3896 ± 0.0315	0.3674 ± 0.0256	0.4317 ± 0.0182	0.4062 ± 0.0139
DeepTTA	0.4743 ± 0.0092	0.4568 ± 0.0065	0.4286 ± 0.0237	0.4121 ± 0.0173	0.4872 ± 0.0131	0.4586 ± 0.0096	0.4524 ± 0.0145	0.4258 ± 0.0108
BANDRP	0.4395 ± 0.0264	0.4216 ± 0.0191	0.4697 ± 0.0086	0.4524 ± 0.0069	0.4431 ± 0.0198	0.4198 ± 0.0143	0.4863 ± 0.0097	0.4589 ± 0.0071
PASO	0.4631 ± 0.0128	0.4477 ± 0.0087	0.3978 ± 0.0301	0.3826 ± 0.0244	0.4179 ± 0.0272	0.3953 ± 0.0211	0.3985 ± 0.0296	0.3747 ± 0.0232
GraphTCDR	0.4818 ± 0.0073	0.4664 ± 0.0054	0.4473 ± 0.0152	0.4309 ± 0.0117	0.4968 ± 0.0084	0.4707 ± 0.0062	0.4691 ± 0.0123	0.4436 ± 0.0094
MTEGDRP	0.4452 ± 0.0189	0.4293 ± 0.0136	0.4762 ± 0.0109	0.4581 ± 0.0081	0.4615 ± 0.0167	0.4382 ± 0.0124	0.4388 ± 0.0205	0.4135 ± 0.0159
MulDR	**0.4936 ± 0.0061**	**0.4785 ± 0.0042**	**0.4889 ± 0.0057**	**0.4727 ± 0.0038**	**0.5174 ± 0.0068**	**0.4906 ± 0.0047**	**0.5286 ± 0.0052**	**0.4997 ± 0.0035**

Supplementary Table S5 Statistical comparisons on the main benchmark

Cells report paired t statistic and Holm-adjusted P value in parentheses for MulDR minus the corresponding baseline over five matched random-seed runs. Holm correction was applied across the seven baseline comparisons separately for each metric.

Baseline	RMSE	MAE	R^2	PCC	SCC
DeepCDR	-16.77 (4.40×10^{-4})	-22.98 (1.15×10^{-4})	13.54 (6.56×10^{-4})	9.98 (1.70×10^{-3})	5.78 (0.0222)
GraphDRP	-16.81 (4.40×10^{-4})	-21.39 (1.15×10^{-4})	14.51 (6.56×10^{-4})	11.54 (1.61×10^{-3})	6.64 (0.0178)
DeepTTA	-16.35 (4.40×10^{-4})	-26.30 (8.69×10^{-5})	17.00 (4.91×10^{-4})	13.54 (1.11×10^{-3})	5.52 (0.0222)
BANDRP	-22.76 (1.55×10^{-4})	-20.00 (1.15×10^{-4})	15.56 (5.97×10^{-4})	13.82 (1.11×10^{-3})	5.63 (0.0222)
PASO	-15.28 (4.40×10^{-4})	-23.56 (1.15×10^{-4})	13.28 (6.56×10^{-4})	8.35 (2.25×10^{-3})	5.67 (0.0222)
GraphTCDR	-9.97 (5.69×10^{-4})	-12.14 (2.64×10^{-4})	11.03 (6.56×10^{-4})	7.74 (2.25×10^{-3})	2.83 (0.0474)
MTEGDRP	-15.75 (4.40×10^{-4})	-21.38 (1.15×10^{-4})	14.29 (6.56×10^{-4})	11.39 (1.61×10^{-3})	6.73 (0.0178)

Supplementary Table S6 Statistical comparisons under cold start evaluation

Cells report paired t statistic and Holm-adjusted P value in parentheses for MulDR minus the corresponding baseline over five matched random-seed runs.

Baseline	Drug RMSE	Drug PCC	Cell-line RMSE	Cell-line PCC
DeepCDR	-25.41 (5.70×10^{-5})	11.80 (1.77×10^{-3})	-14.10 (4.40×10^{-4})	5.59 (0.0153)
GraphDRP	-35.30 (1.92×10^{-5})	11.29 (1.77×10^{-3})	-42.91 (1.06×10^{-5})	6.99 (0.0132)
DeepTTA	-7.02 (4.34×10^{-3})	4.42 (0.0230)	-7.80 (2.91×10^{-3})	5.03 (0.0153)
BANDRP	-15.34 (3.16×10^{-4})	9.06 (2.47×10^{-3})	-85.35 (7.91×10^{-7})	12.83 (1.49×10^{-3})
PASO	-42.21 (1.13×10^{-5})	17.80 (4.10×10^{-4})	-17.33 (2.60×10^{-4})	6.36 (0.0153)
GraphTCDR	-4.79 (8.72×10^{-3})	2.57 (0.0619)	-6.21 (3.42×10^{-3})	3.99 (0.0162)
MTEGDRP	-77.97 (1.14×10^{-6})	10.07 (2.19×10^{-3})	-19.08 (2.22×10^{-4})	6.40 (0.0153)

Supplementary Table S7 Computational resources and efficiency

Item	Value
GPU	NVIDIA GeForce RTX 3090 24 GB
CPU	Dual AMD EPYC 7642 processors
System memory	755 GiB
Software	Linux 5.15.0-136; Python 3.10.8; PyTorch 2.1.2+cu121; CUDA 12.1; cuDNN 8.9.2
Trainable parameters	21.41 million
Training validation test pairs	65,175 / 8,146 / 8,146
Batch size	128
Maximum actual epochs	25 / 19
Best checkpoint epoch	17, selected by validation RMSE from the EMA checkpoint
Mean epoch wall-clock time	14.59 min, including training and validation
Training-loop time	4 h 37 min
End-to-end runtime	4 h 40 min
Peak allocated GPU memory	4.75 GB
Test inference time	95.39 s for 8,146 pairs
Inference time per pair	11.71 ms

Supplementary Figure S1 Parameter sensitivity

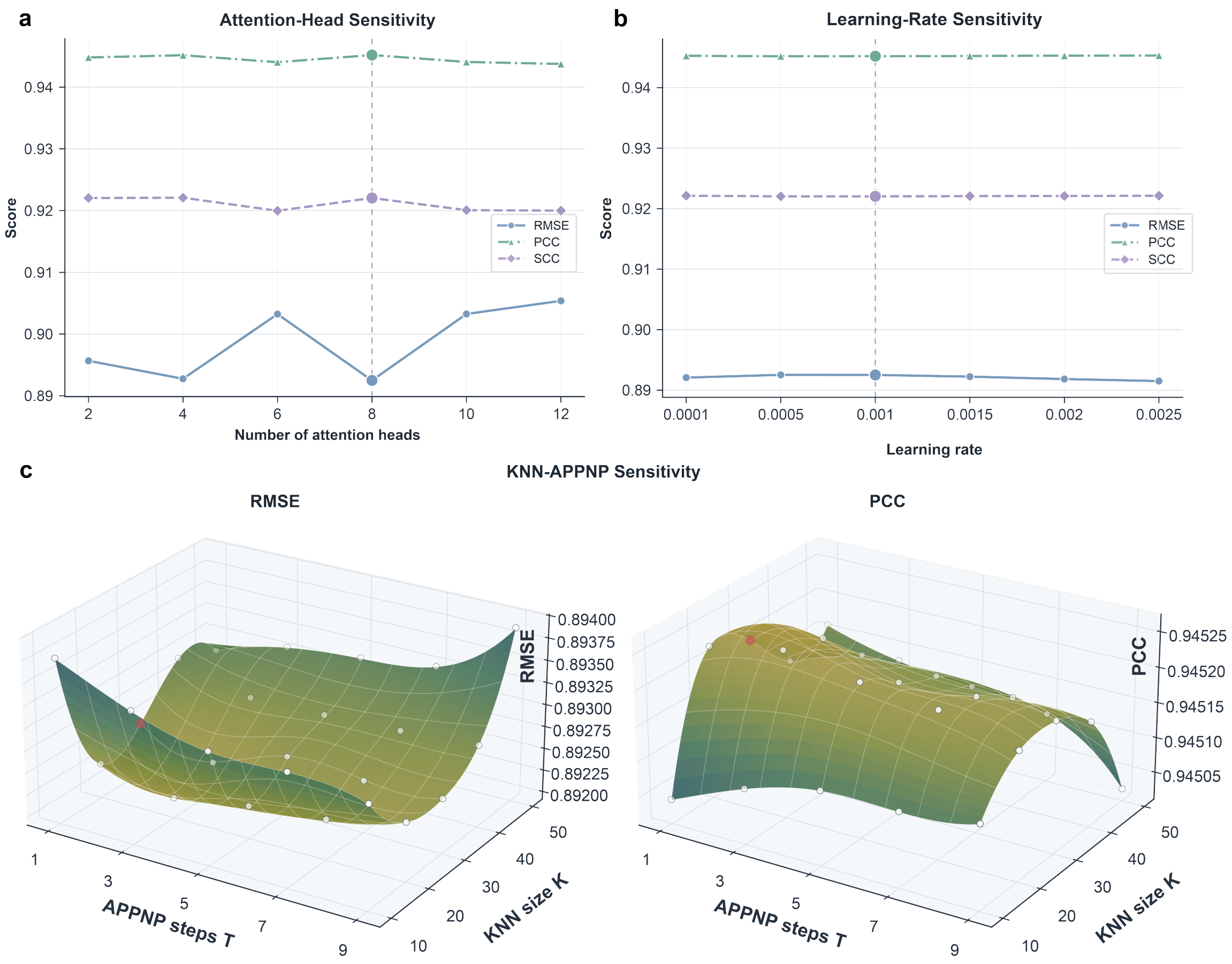

Figure S1. Parameter sensitivity analysis of MulDR. (a) Effect of the number of attention heads. (b) Effect of learning rate. (c) Joint effect of KNN neighborhood size and APPNP propagation steps on RMSE and PCC.

Supplementary Figure S2 Motif and Morgan drug representations

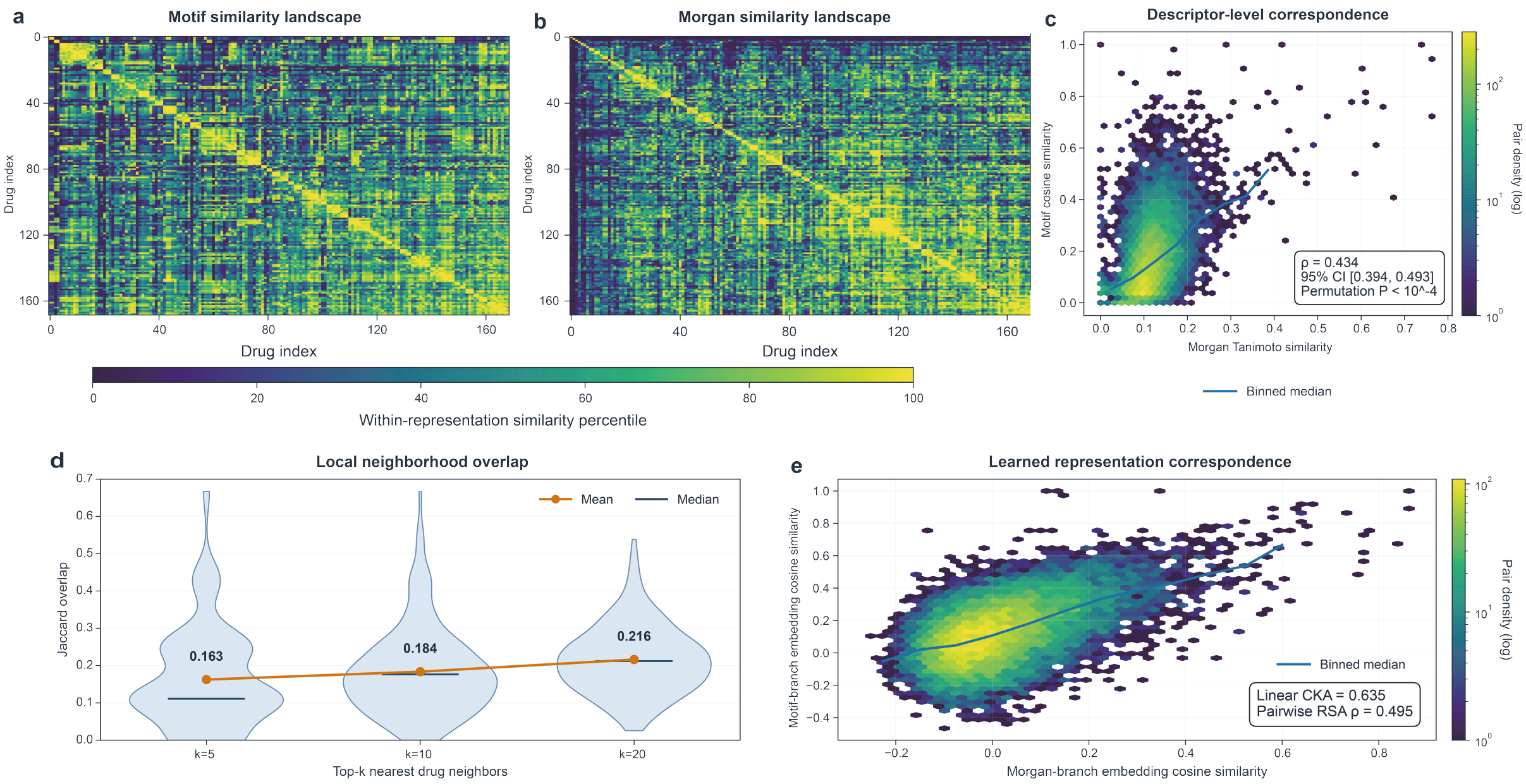

Figure S2. Comparison of motif and Morgan drug representations across 169 drugs. (a) Motif similarity landscape. (b) Morgan similarity landscape. (c) Descriptor-level correspondence between motif cosine similarity and Morgan Tanimoto similarity. (d) Top-k neighborhood overlap between the two representations. (e) Correspondence between the learned motif-branch and Morgan-branch embeddings.

Supplementary Figure S3 Drug-conditioned view weights

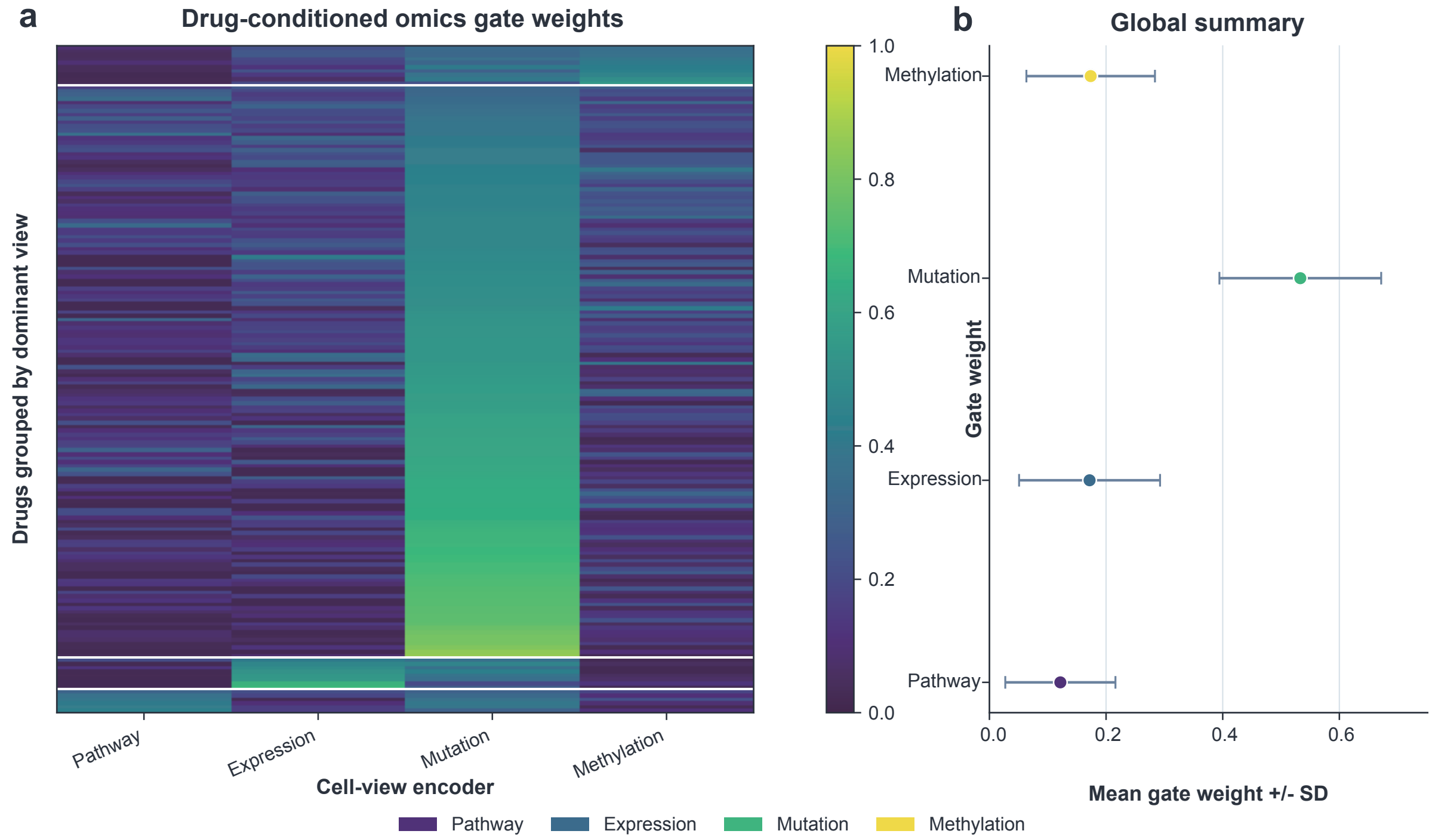

Figure S3. Drug-conditioned omics-view weights across 169 drugs. (a) Gate-weight distributions over pathway, expression, mutation, and DNA-methylation relation spaces, with drugs grouped by dominant view. (b) Mean gate weight and standard deviation across drugs for each omics view.
